# A flavonoid-rich fraction of *Euphorbia peplus* attenuates hyperglycemia, insulin resistance, and oxidative stress in a type 2 diabetes rat model

**DOI:** 10.3389/fphar.2023.1204641

**Published:** 2023-06-16

**Authors:** Reem S. Alruhaimi, Gomaa Mostafa-Hedeab, Maisa Siddiq Abduh, Albandari Bin-Ammar, Emad H. M. Hassanein, Emadeldin M. Kamel, Ayman M. Mahmoud

**Affiliations:** ^1^ Department of Biology, College of Science, Princess Nourah Bint Abdulrahman University, Riyadh, Saudi Arabia; ^2^ Pharmacology Department, Medical College, Jouf University, Sakaka, Saudi Arabia; ^3^ Pharmacology Department, Faculty of Medicine, Beni-Suef University, Beni-Suef, Egypt; ^4^ Immune Responses in Different Diseases Research Group, Department of Medical Laboratory Sciences, Faculty of Applied Medical Sciences, King Abdulaziz University, Jeddah, Saudi Arabia; ^5^ Center of Excellence in Genomic Medicine Research, King Abdulaziz University, Jeddah, Saudi Arabia; ^6^ Department of Clinical Nutrition, College of Applied Medical Sciences, University of Hail, Hail, Saudi Arabia; ^7^ Department of Pharmacology and Toxicology, Faculty of Pharmacy, Al-Azhar University, Assiut, Egypt; ^8^ Chemistry Department, Faculty of Science, Beni-Suef University, Beni-Suef, Egypt; ^9^ Department of Life Sciences, Faculty of Science and Engineering, Manchester Metropolitan University, Manchester, United Kingdom; ^10^ Physiology Division, Zoology Department, Faculty of Science, Beni-Suef University, Beni-Suef, Egypt

**Keywords:** *Euphorbia*, diabetes, insulin resistance, oxidative stress, inflammation

## Abstract

**Background:** Type 2 diabetes (T2D) is a metabolic disorder characterized by insulin resistance (IR) and hyperglycemia. Plants are valuable sources of therapeutic agents for the management of T2D. *Euphorbia peplus* has been widely used as a traditional medicine for the treatment of various diseases, but its beneficial role in T2D has not been fully explored.

**Methods:** The anti-diabetic efficacy of *E. peplus* extract (EPE) was studied using rats with T2D induced by high-fat diet (HFD) and streptozotocin (STZ). The diabetic rats received 100, 200, and 400 mg/kg EPE for 4 weeks.

**Results:** Phytochemical fractionation of the aerial parts of *E. peplus* led to the isolation of seven known flavonoids. Rats with T2D exhibited IR, impaired glucose tolerance, decreased liver hexokinase and glycogen, and upregulated glycogen phosphorylase, glucose-6-phosphatase (G-6-Pase), and fructose-1,6-bisphosphatase (F-1,6-BPase). Treatment with 100, 200, and 400 mg/kg EPE for 4 weeks ameliorated hyperglycemia, IR, liver glycogen, and the activities of carbohydrate-metabolizing enzymes. EPE attenuated dyslipidemia, serum transaminases, tumor necrosis factor (TNF)-α, interleukin (IL)-1β and liver lipid accumulation, nuclear factor (NF)-κB p65, and lipid peroxidation, nitric oxide and enhanced antioxidants. All EPE doses upregulated serum adiponectin and liver peroxisome proliferator-activated receptor γ (PPARγ) in HFD/STZ-induced rats. The isolated flavonoids showed *in silico* binding affinity toward hexokinase, NF-κB, and PPARγ.

**Conclusion:** *E. peplus* is rich in flavonoids, and its extract ameliorated IR, hyperglycemia, dyslipidemia, inflammation and redox imbalance, and upregulated adiponectin and PPARγ in rats with T2D.

## 1 Introduction

Diabetes mellitus (DM) is a common metabolic disorder associated with several complications, including nephropathy, neuropathy, and cardiomyopathy. This disorder includes type 1 (T1DM) and type 2 (T2DM) forms of the disease where T1DM is characterized by insulin insufficiency, whereas insulin resistance (IR) is the characteristic feature of T2DM. Both insulin deficiency and IR lead to the accumulation of glucose in the blood (hyperglycemia) (American Diabetes Association, 2021). DM is a fast-increasing disease worldwide, and the number of patients with diabetes is expected to reach 700 million by 2045 (Saeedi et al., 2019). T2DM is the most common form of the disease characterized by hyperglycemia and IR (Kahn et al., 2014). IR increases the risk of hypertension, dyslipidemia, and atherosclerosis (Guzik and Cosentino, 2018). Oxidative stress (OS) and inflammation mediated by excess reactive oxygen species (ROS) and inflammatory mediators produced under hyperglycemic conditions are implicated in the pathophysiology of DM and its complications (Mahmoud et al., 2012). Excess ROS can damage cellular macromolecules and work in concert with inflammatory mediators to provoke cell death. ROS and inflammatory mediators impair insulin signaling by provoking *β*-cell death, alter peripheral glucose uptake, and increase gluconeogenesis (Jheng et al., 2012). Therefore, mitigation of OS and inflammation could be beneficial to prevent IR and hyperglycemia in T2DM.

Plants of the genus *Euphorbia* include numerous known species with chemical diversity and multiple biological and commercial uses (Shi et al., 2008). The latex of plants of the family *Euphorbiaceae* was acknowledged for its various phytoconstituents that possess both commercial and pharmacological importance such as triterpene alcohols (Giner and Schroeder, 2015). Because of its toxic nature and unpleasantness, the latex protects the plants against the attack of animals (Al-Sultan and Hussein, 2006). Steroids, flavonoids, sesquiterpenoids, glycerols, and cerebrosides are among the phytoconstituents reported in plants of the genus *Euphorbia* (Shi et al., 2008; Kamel et al., 2022). With this rich content, *Euphorbia* plants found their way to be employed in folkloric medicine to treat migraine, intestinal parasites, gonorrhea, and skin disorders (Singla and Kamla, 1990), and studies have reported their wound-healing potential (Pattanaik et al., 2014; Ahmed et al., 2016). Recent work from our laboratory revealed the inhibitory activity of *E. peplus* on xanthine oxidase (XO) and hyperuricemia in rats (Kamel et al., 2022). Other studies showed the possible beneficial effects of *E. royleana* stem extract (Zafar et al., 2021) and *E. hirta* flower extract (Kumar et al., 2010) in rats with streptozotocin (STZ)- and alloxan-induced diabetes, respectively. These studies revealed the ability of *E. royleana* and *E. hirta* to ameliorate hyperglycemia and oxidative damage. Another recent study highlighted the anti-hyperglycemic effect of *E. helioscopia* methanolic extract in sucrose-supplemented rats (Mustafa et al., 2022). Owing to the promising therapeutic value of plants of this genus, this study explored the phytochemical constituents and the effect of *E. peplus* extract (EPE) on hyperglycemia, IR, OS, and inflammation in rats with T2D induced by high-fat diet (HFD) and STZ.

## 2 Materials and methods

### 2.1 Phytochemical investigation

#### 2.1.1 General

Proton nuclear magnetic resonance (^1^HNMR) and ^13^CNMR (500 MHz and 125 MHz, respectively) spectra were recorded on the Bruker AV-500 spectrometer using TMS as an internal standard. The optical rotation of isolated flavonoids was obtained using a Rudolph Autopol III polarimeter. Ultraviolet (UV) spectral data were measured using the Shimadzu UV-vis 160i spectrophotometer, and the HREIMS and EIMS spectral data were recorded using the Finnigan MAT TSQ 700 mass spectrometer. Infrared spectral data were obtained through KBr pellets on the Shimadzu FTIR-8400 instrument.

#### 2.1.2 Plant collection, extraction, and isolation

The plant was collected from Beni Suef Governorate in March 2021 and identified by a taxonomist and a voucher specimen (EP-038021-2) was stored. The aerial parts (2.75 kg) were extracted four times using 70% acetone followed by the removal of the solvent under reduced pressure, resulting in 904 g of extract. Thereafter, the extract was dissolved in water and successively partitioned using chloroform, ethyl acetate (EA), and *n*-butanol (3L x 2, each). The EA fraction (69.7 g) was subjected to chromatographic fractionation over a silica gel column (120 × 4 cm, 1.1 kg) and eluted with dichloromethane (DCM)/acetone mixture of increasing polarity. To track the movement of the bands along the column and to regulate the collection of fractions, a UV lamp was employed. A total of 22 fractions were collected and combined into seven main subfractions (F1–F7) according to their similar thin-layer chromatography (TLC) profiles. Subfraction F3 was chromatographed over silica gel using chloroform–EA of gradient elution to afford nine subfractions (F3.1–F3.9). Subfractions F3.3–F3.7 were combined and applied to the Sephadex LH-20 column eluted with methanol (MeOH):water (50:50→100:0) to give seven TLC-monitored subfractions (E1–E7). Sub-subfractions (E2–E5) were combined and purified over a Sephadex LH-20 column eluted with MeOH to yield the purified compounds 2 (22 mg), 3 (17 mg), and 4 (14 mg). Subfraction F4 was fractionated over a polyamide 6S column eluted with the MeOH–water solvent mixture of increasing polarity to afford eleven subfractions (F4.1–F4.11). Subfraction F4.6 was purified over the Sephadex LH-20 column eluted with MeOH to yield purified compound 1 (23 mg). Subfractions F4.8–F4.10 were combined and re-chromatographed using the Sephadex LH-20 column eluted with MeOH to give compound 5 (19 mg). Subfraction F5 was partitioned by means of the Sephadex LH-20 column using MeOH–water (2:8, 3:7→10:0) to afford six subfractions (F5.1–F5.6). Compound 6 (23 mg) was obtained from the chromatographic fractionation of F5.3–F5.5 over two consecutive Sephadex LH-20 columns using 30% MeOH as an eluent. Subfraction F7 was subjected to silica gel column chromatography eluted with the solvent system chloroform–MeOH–water (lower layer, 28:9:6 and 6:3:1) to yield five subfractions (F7.1–F7.5). Compound 7 (24 mg) was obtained from the recombination and purification of subfractions F7.1–F7.3 over Sephadex LH-20 and eluted with MeOH (Supplementary Figure S1).

### 2.2 *In vitro* radical-scavenging activity

The RSA activity of EPE was measured using 2,2-diphenyl-1-picrylhydrazyl (DPPH) and 2,2-azinobis (3-ethylbenzothiazoline-6-sulfonic acid) (ABTS) assays following the methods of Brand-Williams et al. (1995) and Re et al. (1999), respectively, using ascorbic acid as a standard.

### 2.3 Experimental animals and treatments

Male Wistar rats weighing 170–190 g were included in this investigation. The rats were maintained under standard conditions of temperature (23°C ± 1°C) and humidity (50%–60%) on a 12-h light/dark cycle with free access to food and water. The animal study protocol was approved by the Research Ethics Committee of Al-Azhar University (ZA-AS/PH/18/C/2023). The rats received a normal diet and a single intraperitoneal (i.p.) injection of freshly prepared citrate buffer (pH 4.5) to serve as a control. Other rats were fed a HFD (58% fat, 17% carbohydrate, and 25% protein) for 28 days and received a single i.p. dose of STZ (35 mg/kg; Sigma, United States) dissolved in freshly prepared citrate buffer (pH 4.5) to induce T2D (Germoush et al., 2019). After 7 days, T2D was confirmed by measuring blood glucose (BG) for 2 h after supplementing the overnight fasted rats with 3 g/kg glucose orally. The rats exhibited BG higher than 250 mg/kg were included in the investigation.

To investigate the antidiabetic effects of the EA fraction of *E. peplus* extract (EPE; dissolved in 0.5% carboxymethyl cellulose (CMC) as a vehicle), 30 diabetic and 12 normal rats were allocated into seven groups (*n* = 6) as follows:

Group I (control): received 0.5% CMC.
Group II (EPE): received 400 mg/kg EPE.
Group III (diabetic): received 0.5% CMC.
Group IV (diabetic + 100 mg/kg EPE): received 100 mg/kg EPE.
Group V (diabetic + 200 mg/kg EPE): received 200 mg/kg EPE.
Group VI (diabetic + 400 mg/kg EPE): received 400 mg/kg EPE.
Group VII (diabetic + PIO): received 10 mg/kg of the antidiabetic pioglitazone (PIO) (Abd El-Twab et al., 2016).

EPE, 0.5% CMC, and PIO were supplemented orally for 4 weeks. A day before the end of the experiment, the rats were fasted overnight and then supplemented with 3 g/kg glucose solution, and the blood was collected from the tail vein over 2 h for the determination of BG using a Spinreact (Spain) kit (Trinder, 1969). At the end of treatments, the animals were euthanized under ketamine anesthesia (100 mg/kg i.p.), and blood and liver samples were collected. Serum was separated following centrifugation of blood, and samples from the liver were homogenized in Tris-HCl buffer (pH = 7.4). Other samples were fixed in 10% neutral buffered formalin (NBF) or stored at −80°C.

### 2.4 Biochemical assays

Serum insulin, transaminases (ALT and AST), adiponectin and cytokines (TNF-α and IL-1β) were determined using kits from RayBiotech (United States), Spinreact (Spain), and R&D Systems (United States), respectively. NF-kB p65 in liver homogenate was determined using the kit from R&D Systems (United States). All assays were performed according to the manufacturers’ instructions.

The homeostasis model assessment of IR (HOMA-IR) was calculated as previously described by Haffner (2000) using the following equation:

$$HOMA-IR=\frac{Fasting\;insulin\;\left(\frac{\mu U}{ml}\right)\times Fasting\;glucose\;\left(\frac{mmol}{L}\right)}{22.5}.$$

Liver glycogen content was determined as previously described (Seifter and Dayton, 1950). Liver homogenate was centrifuged, and the clear supernatant was used to assess the activities of hexokinase (Brandstrup et al., 1957), G-6-Pase (Koide and Oda, 1959), F-1,6-BPase (Freedland and Harper, 1959), and glycogen phosphorylase (Stalmans and Hers, 1975). Malondialdehyde (MDA) (Ohkawa et al., 1979), nitric oxide (NO) (Green et al., 1982), reduced glutathione (GSH) (Beutler et al., 1963), and the activities of superoxide dismutase (SOD) (Marklund and Marklund, 1974), catalase (CAT) (Aebi, 1984), and glutathione peroxidase (GPx) (Flohé and Günzler, 1984) were determined in the supernatant of the homogenized liver. Liver triglycerides (TGs) and cholesterol were assayed using Spinreact (Spain) kits after extracting the lipids using chloroform/MeOH mixture (2:1, v/v) as descried by Folch *et al* (1957). Serum TG, total cholesterol (TC), and high-density lipoprotein (HDL)-C were assayed using kits from Spinreact (Spain). Low-density lipoprotein (LDL)-C, very low-density lipoprotein (vLDL)-C, and atherogenic index of plasma (AIP) were calculated according to the following equations:

vLDL.C=TG/5,

$$LDL.C=TC-\left(HDL.C+vLDL.C\right),$$

$$AIP=\mathrm{Log}\;\left(\frac{TG}{HDL}\right).$$

### 2.5 Histopathological study

Samples from the liver fixed in 10% NBF for 24 h were dehydrated, cleared, and embedded in paraffin wax. Sections of 5 μm thickness were cut for routine staining with hematoxylin and eosin (H&E) (Bancroft and Gamble, 2008) and examined under a light microscope.

### 2.6 Quantitative real-time polymerase chain reaction

To determine the changes in PPARγ mRNA, RNA was isolated from the frozen liver using TRIzol and quantified, and samples with OD260/280 ≥ 1.8 were reverse-transcribed into cDNA using a cDNA synthesis kit (Thermo Scientific, United States). cDNA amplification was carried out using SYBR Green Master Mix (Thermo Scientific, United States), and the primers used in the qRT-PCR experiment are listed in Supplementary Table S1. The Ct values were analyzed by using the 2^−ΔΔCt^ method (Livak and Schmittgen, 2001).

### 2.7 *In silico* molecular docking

The binding of *E. peplus* compounds with hexokinase II (PDB ID: 2NZT), NF-κB–DNA complex (PDB ID 1LE9), and PPARγ (PDB ID: 2PRG) was investigated as previously reported (Supplementary Material) (Sami et al., 2022; Abduh et al., 2023).

### 2.8 Statistical analysis

The obtained results are presented as mean ± standard deviation (SD), and all statistical comparisons were made using one-way ANOVA followed by *post hoc* Tukey’s test on GraphPad Prism 8 software. *p*-values < 0.05 were considered statistically significant.

## 3 Results

### 3.1 Phytochemical investigation and *in vitro* RSA

**FIGURE 1:**
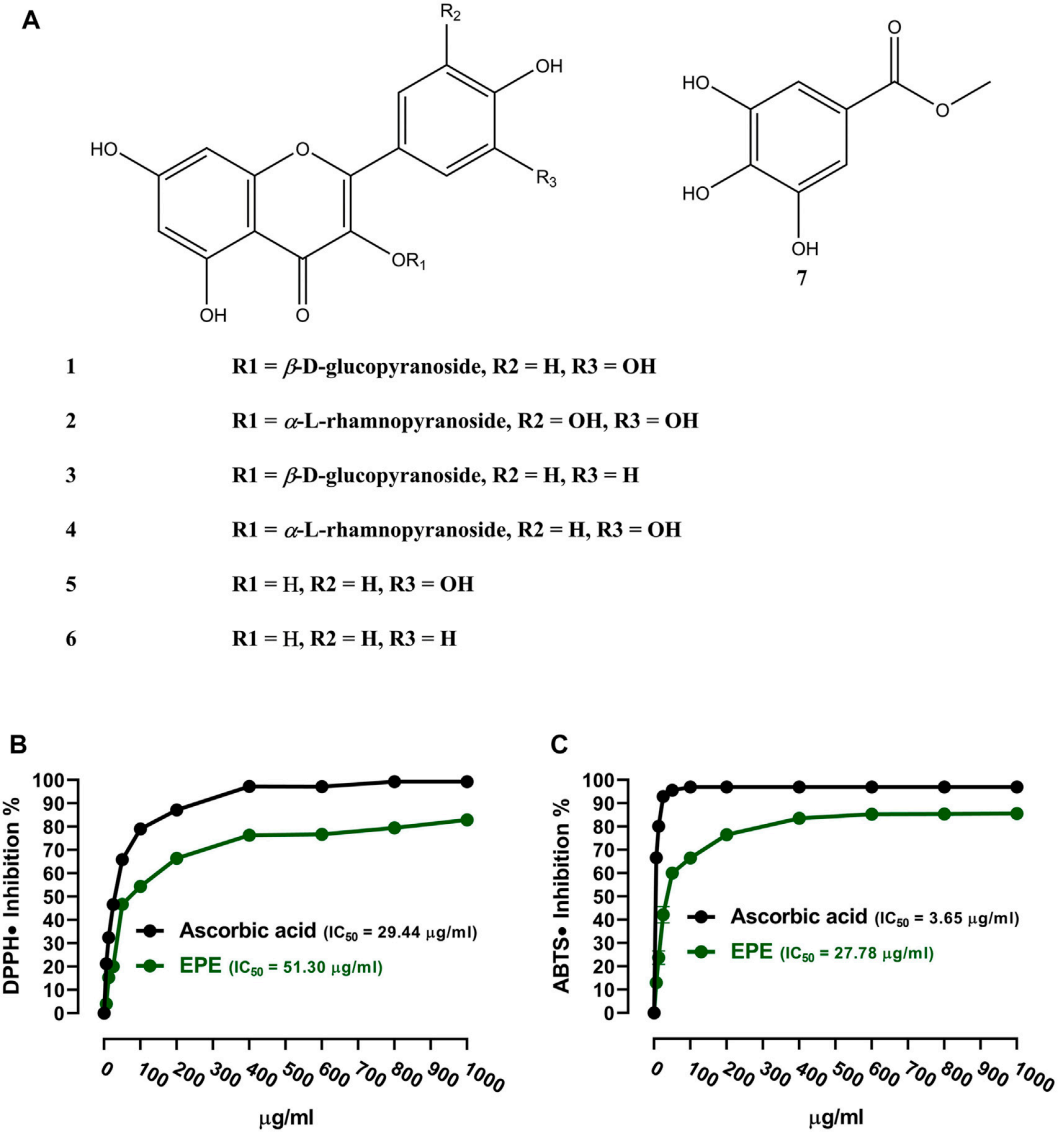
**(A)** Chemical structure of the isolated compounds. **(B, C)** DPPH and ABTS radical-scavenging activities of EPE. Data are mean ± SD (N = 3).

The analysis of the EA fraction of *E. peplus* led to the isolation of seven known flavonoids. Structures of isolated compounds (**1**-**7**) were elucidated based on spectroscopic data (Supplementary Figures S2–15) and by comparison with those previously reported. The isolated flavonoids (Figure 1A) were identified as isoquercetin (**1**) (Han et al., 2004), myricitrin (**2**) (Fu et al., 2013), astragalin (**3**) (Wei et al., 2011), quercitrin (**4**) (Tatsis et al., 2007), quercetin (**5**) (Mabry et al., 1970), kaempferol (**6**) (Mabry et al., 1970; Elsayed et al., 2020), and methyl gallate **(7)** (Ma et al., 2005). The *in vitro* RSA showed a concentration-dependent antioxidant activity of EPE against DPPH (Figure 1C) and ABTS (Figure 1D) radicals with IC_50_ values of 51.30 and 27.78 μg/ml, respectively.

### 3.2 EPE ameliorates glucose intolerance and IR in diabetic rats

**FIGURE 2:**
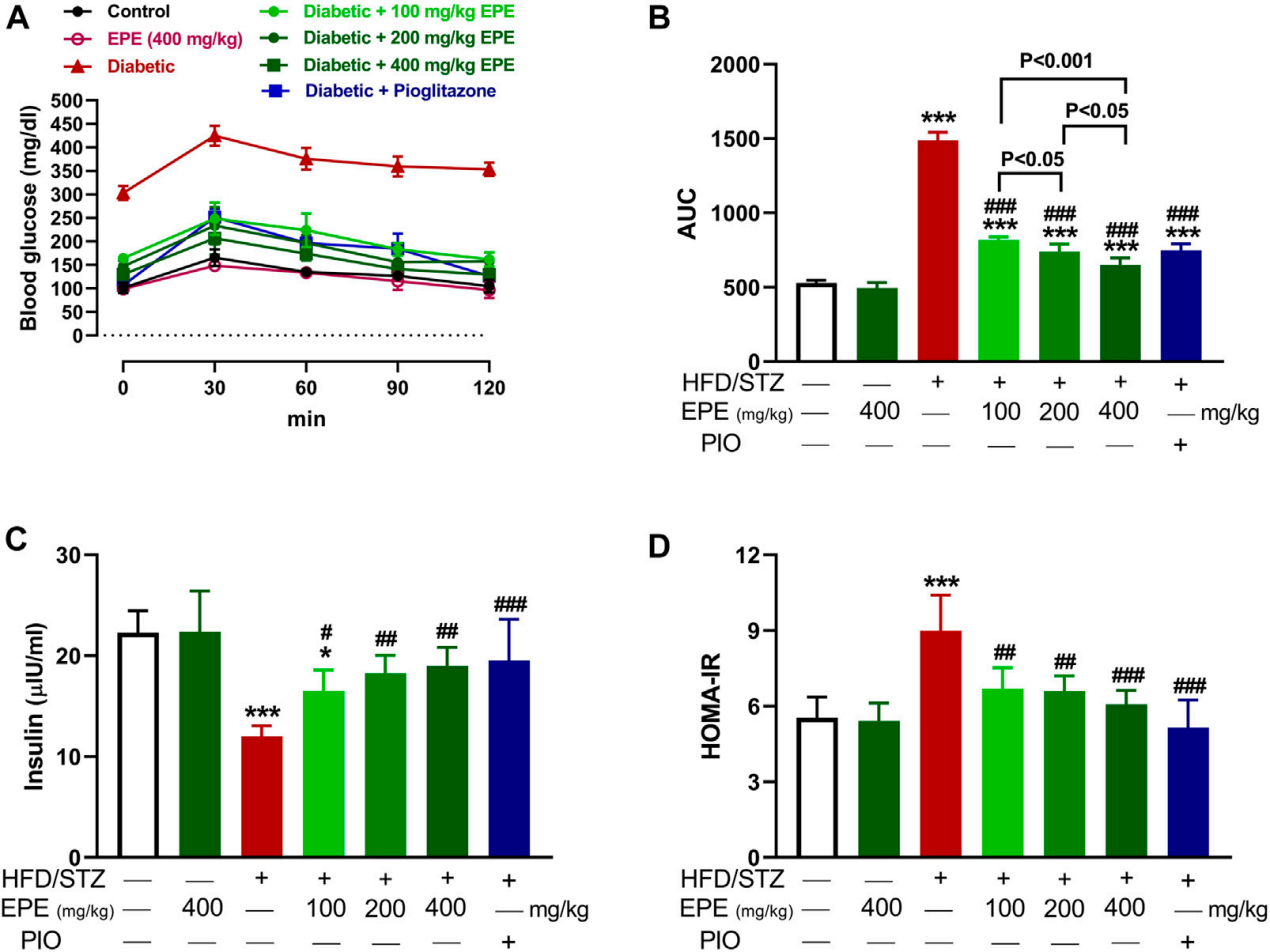
EPE ameliorated glucose intolerance **(A and B)**, serum insulin **(C)**, and HOMA-IR **(D)** in diabetic rats. Data are mean ± SD (*n* = 6). *p < 0.05 and ***p < 0.001 vs. control. #*p* < 0.05, ##*p* < 0.01, and ###*p* < 0.001 vs. diabetic.

OGTT was performed, and insulin was measured to determine the anti-hyperglycemic effect of EPE. The HFD/STZ-induced diabetic rats exhibited significant elevation in BG (Figure 2A, B). Treatment with EPE and PIO effectively ameliorated BG levels in diabetic rats (*p* < 0.001). Insulin was declined in diabetic rats (*p* < 0.001; Figure 2C), and the value of HOMA-IR was elevated (Figure 2D). All doses of EPE effectively alleviated insulin and HOMA-IR (*p* < 0.001). EPE didn’t alter glucose and insulin in normal animals.

### 3.3 EPE modulates carbohydrate-metabolizing enzymes in diabetic rats

**FIGURE 3:**
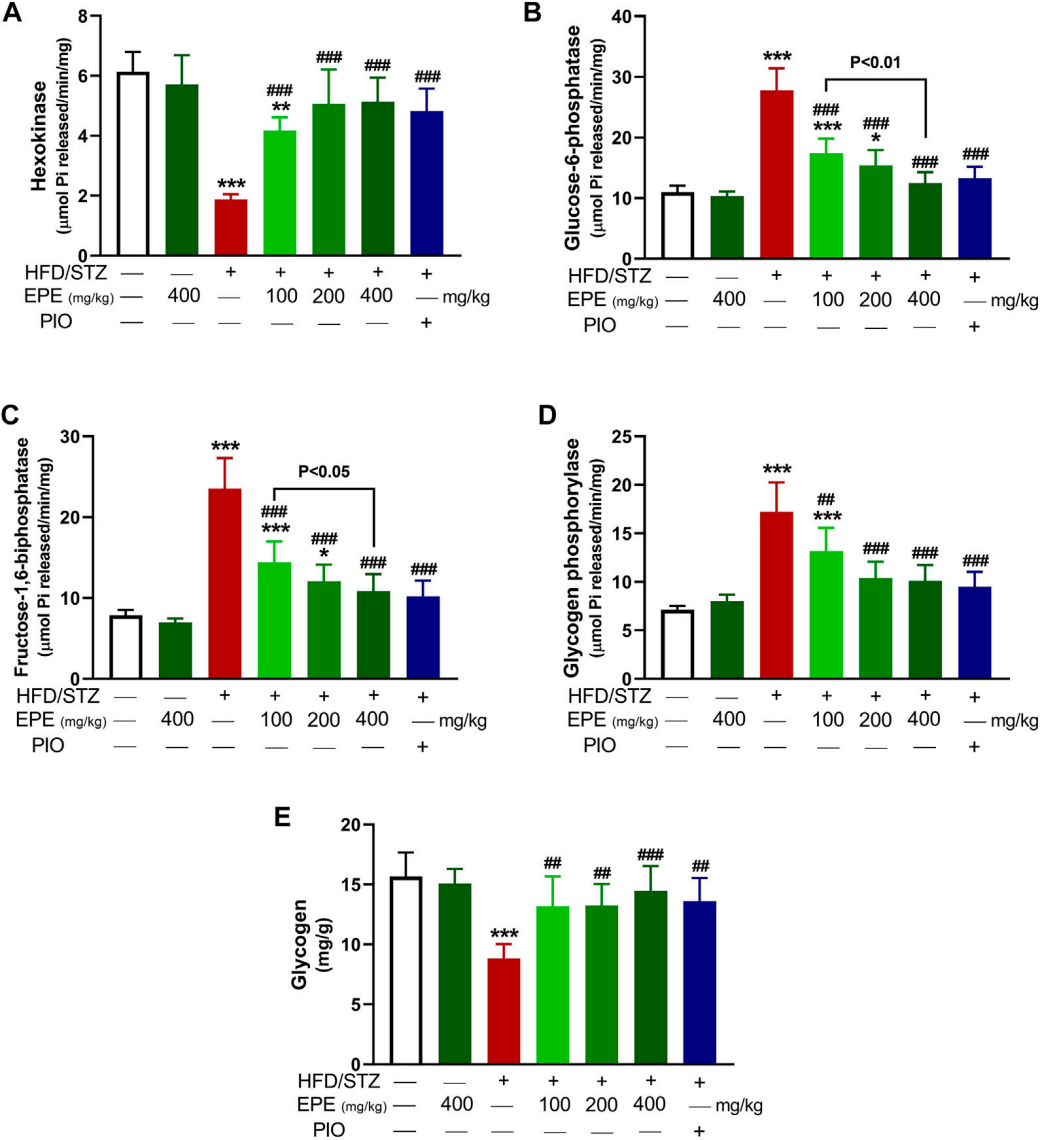
EPE increased hexokinase **(A)**, decreased G-6-Pase **(B)**, F-1,6-BPase **(C)**, and glycogen phosphorylase **(D)** and enhanced glycogen **(E)** in the liver of diabetic rats. Data are mean ± SD (*n* = 6). *p < 0.05, **p < 0.01, and ***p < 0.001 vs. control. ##*p* < 0.01 and ###*p* < 0.001 vs. diabetic.

The activity of hexokinase (Figure 3A) was decreased, and G-6-Pase (Figure 3B), F-1,6-BPase (Figure 3C), and glycogen phosphorylase (Figure 3D) were activated in the diabetic rat liver (*p* < 0.001). Liver glycogen was decreased in diabetic rats as compared to the non-diabetic animals (*p* < 0.001; Figure 3E). EPE remarkably increased hexokinase and glycogen and suppressed other enzymes in diabetic rats.

**FIGURE 4:**
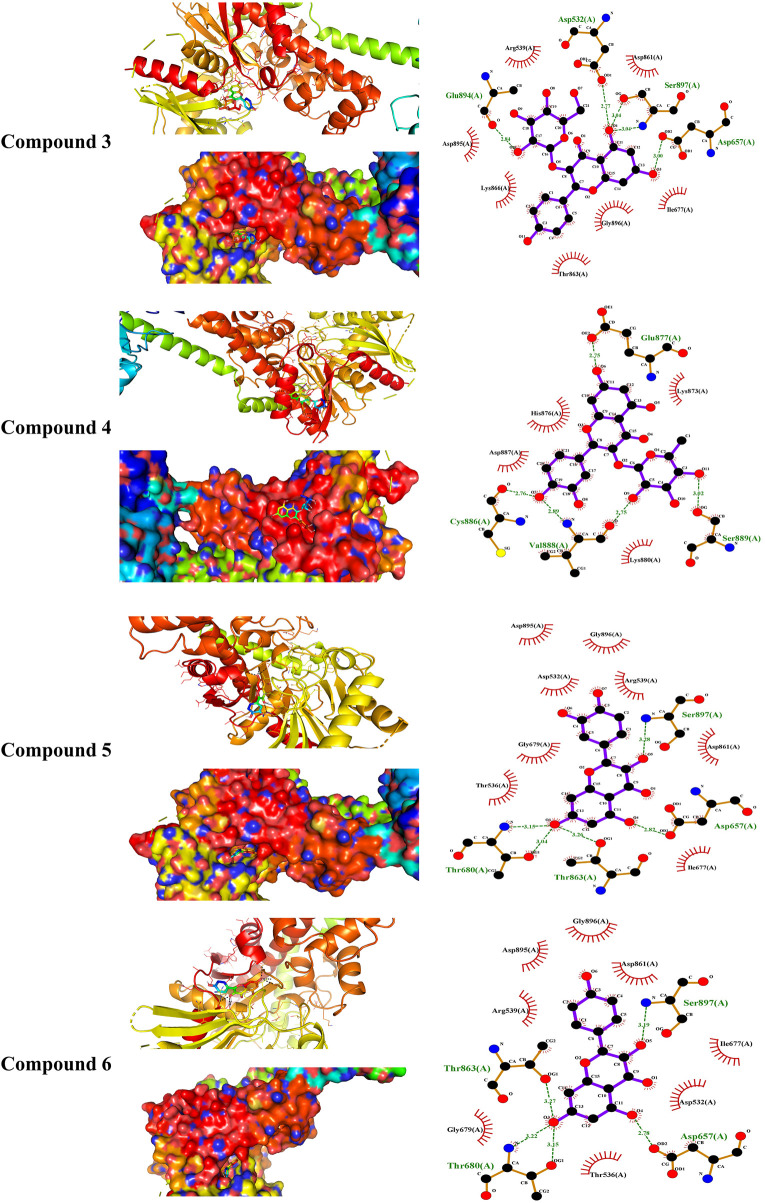
Molecular docking showing the binding modes of compounds **3**, **4**, **5**, and **6** with hexokinase.

MD simulations showed the binding affinity of EPE flavonoids with hexokinase as shown in Table 1 and Figure 4 and Supplementary Figure S16. Compounds **3**, **4**, **5**, and **6** exhibited the lowest binding energy (−7.4, −7.3, −8.1, and −7.8 kcal/mol, respectively) and formed multiple polar bonding and hydrophobic interactions with different amino acid residues (Table 1).

**TABLE 1:** Binding affinities, interacting polar residues, and hydrophobic interactions of the compounds isolated from *E. peplus* with hexokinase.

Compound	Binding energy (kcal/mol)	Polar bond	Hydrophobic interaction
**1**	−6.9	Gly535, Thr536, Gly747, Glu783, Thr784, and Thr863	Thr680, Met748, and Gly780
**2**	−7.0	Leu617 and Gln739	Ala505, Ser506, Ala507, Pro508, Lys510, Pro605, Lys618, Glu708, Ala711, Asp714, Asn715, and Lys738
**3**	−7.4	Asp532, Asp657, Glu864, and Ser897	Arg539, Ile677, Asp861, Thr863, Lys866, Asp895, and Gly896
**4**	−7.3	Glu877, Cys886, Val888, and Ser886	Lys873, His876, Lys880, and Asp887
**5**	−8.1	Asp657, Thr680, Thr863, and Ser897	Asp532, Thr536, Arg539, Ile677, Gly679, Asp861, Asp895, and Gly896
**6**	−7.8	Asp657, Thr680, Thr863, and Ser897	Asp532, Thr536, Arg539, Ile677, Gly679, Asp861, Asp895, and Gly896
**7**	−6.1	Asp532, Thr661, and Ser897	Gly535, Thr536, Ile677, Gly679, Thr680, Asp861, Gly862, and Thr863

### 3.4 EPE ameliorates dyslipidemia and liver lipid accumulation in diabetic rats

**FIGURE 5:**
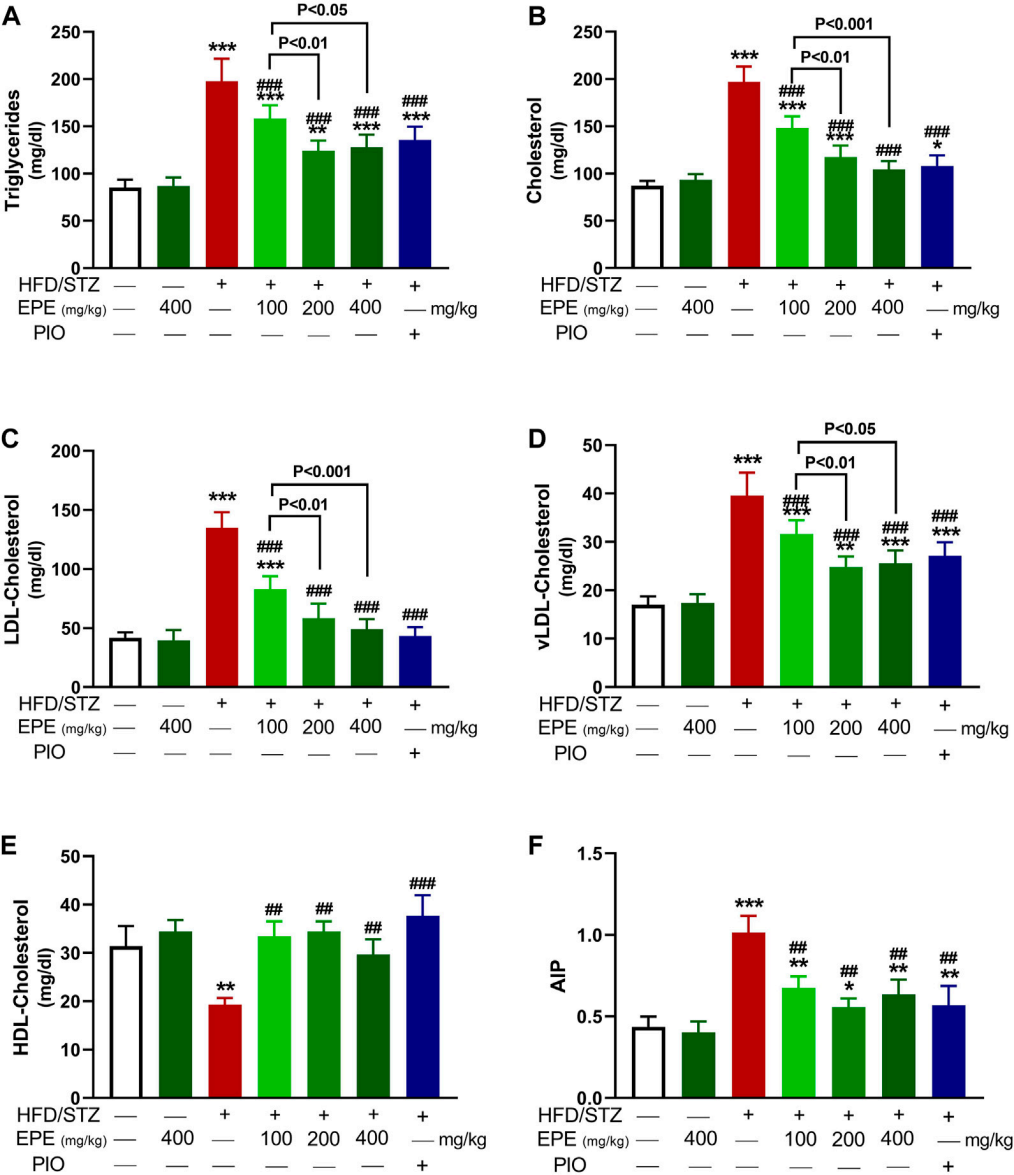
EPE decreased serum TG **(A)**, TC **(B)**, LDL-C **(C)**, vLDL-C **(D)**, and AIP **(F)** and increased HDL-C **(E)** in diabetic rats. Data are mean ± SD (*n* = 6). *p < 0.05, **p < 0.01, and ***p < 0.001 vs. control. ##*p* < 0.01 and ###*p* < 0.001 vs. diabetic.

**FIGURE 6:**
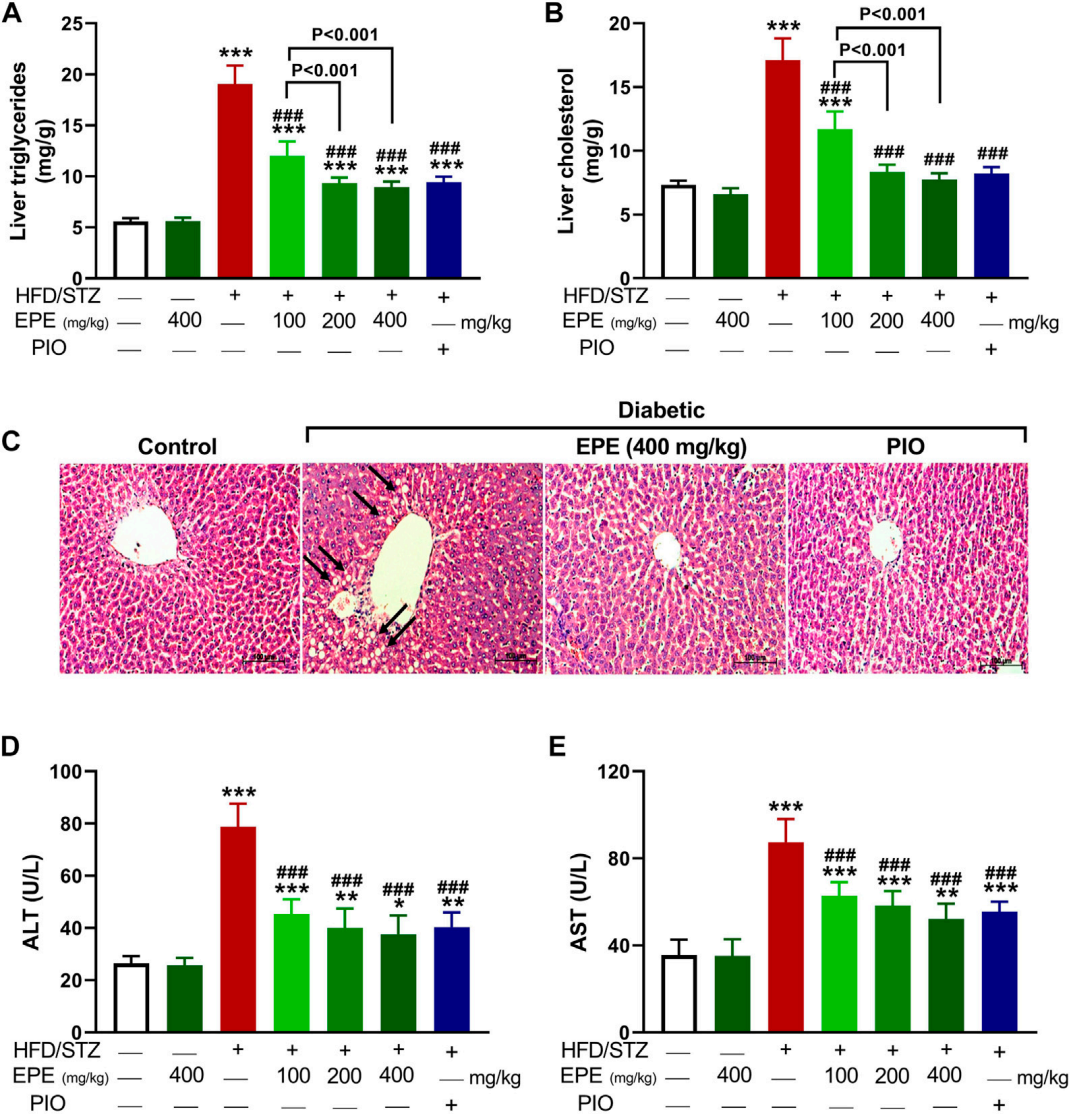
EPE decreased liver TG **(A)** and cholesterol **(B)**, prevented lipid deposition **(C)**, and ameliorated serum ALT **(D)** and AST **(E)** in diabetic rats. Data are mean ± SD (*n* = 6). *p < 0.05, **p < 0.01, and ***p < 0.001 vs. control. ###*p* < 0.001 vs. diabetic.

TG, TC, LDL-C, and vLDL-C were increased in the serum of diabetic rats (*p* < 0.001) as shown in Figures 5A–D. HDL-C was decreased (*p* < 0.01; Figure 5E), and AIP was elevated (*p* < 0.001; Figure 5F) in diabetic animals. All doses of EPE decreased serum lipids and AIP and increased HDL-C in diabetic rats. Dyslipidemia was associated with increased liver TG (Figure 6A) and cholesterol (Figure 6B). Likewise, the stained sections of the liver of diabetic rats revealed the deposition of lipids (Figure 6C) along with increased circulating transaminases (Figures 6D,E; *p* < 0.001). All doses of EPE decreased liver lipids and serum transaminases in diabetic rats.

### 3.5 EPE mitigates oxidative stress in diabetic rats

**FIGURE 7:**
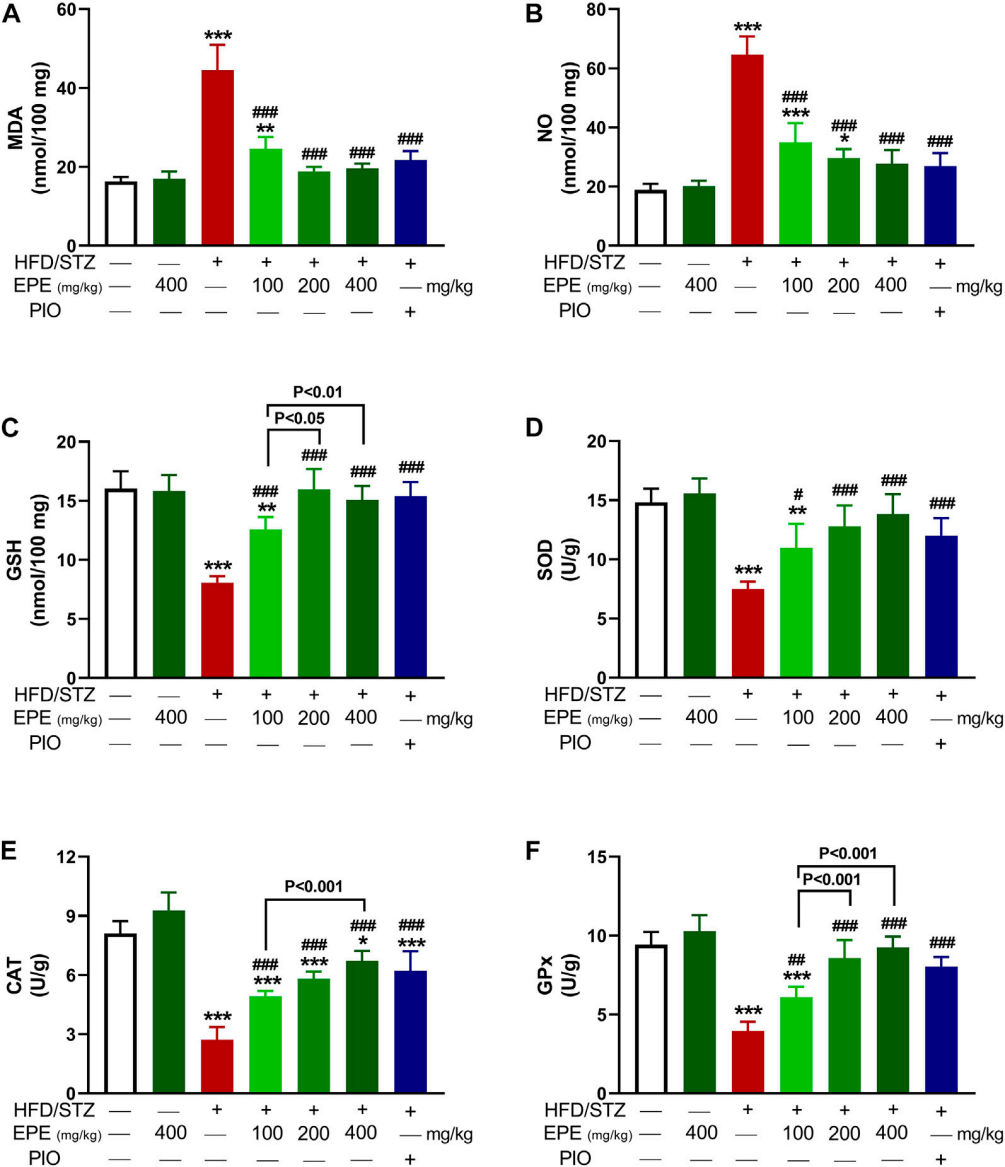
EPE decreased liver MDA **(A)** and NO **(B)** and increased GSH **(C)**, SOD **(D)**, CAT **(E)**, and GPx **(F)** in diabetic rats. Data are mean ± SD (*n* = 6). *p < 0.05, **p < 0.01, and ***p < 0.001 vs. control. #*p* < 0.05, ##*p* < 0.01, and ###*p* < 0.001 vs. diabetic.

MDA and NO were elevated in HFD/STZ-induced rats (*p* < 0.001) as compared to the control rats (Figures 7A,B). In contrast, GSH (Figure 7C), SOD (Figure 7D), CAT (Figure 7E), and GPx (Figure 7F) were decreased in diabetic animals. EPE decreased MDA and NO and increased antioxidants effectively in diabetic rats while showing no effect on normal animals.

### 3.6 EPE attenuates inflammation in diabetic rats

**FIGURE 8:**
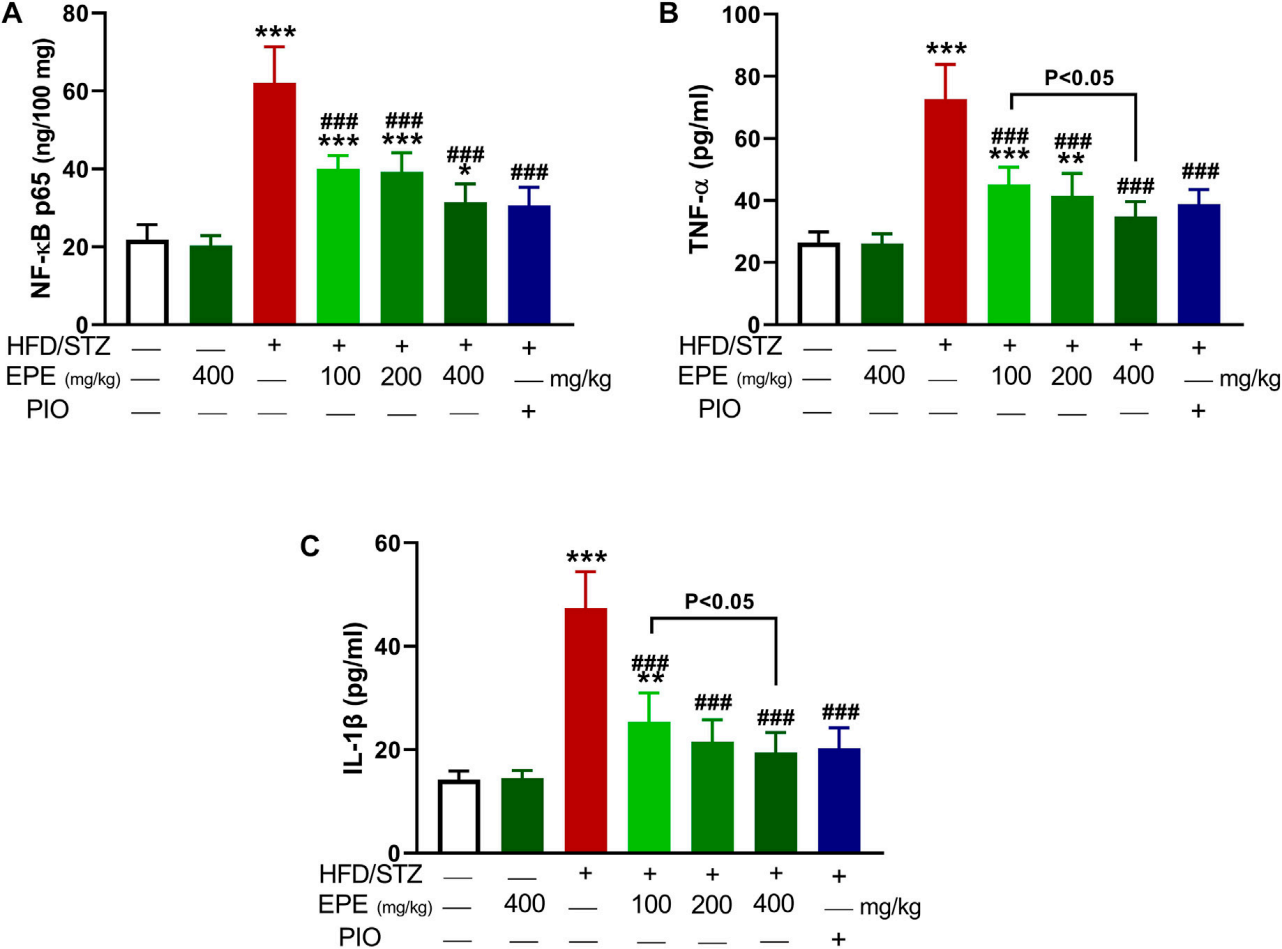
EPE decreased liver NF-kB p65 **(A)** and serum TNF-α **(B)** and IL-1β **(C)** in diabetic rats. Data are mean ± SD (*n* = 6). *p < 0.05, **p < 0.01, and ***p < 0.001 vs. control. ###*p* < 0.001 vs. diabetic.

**FIGURE 9:**
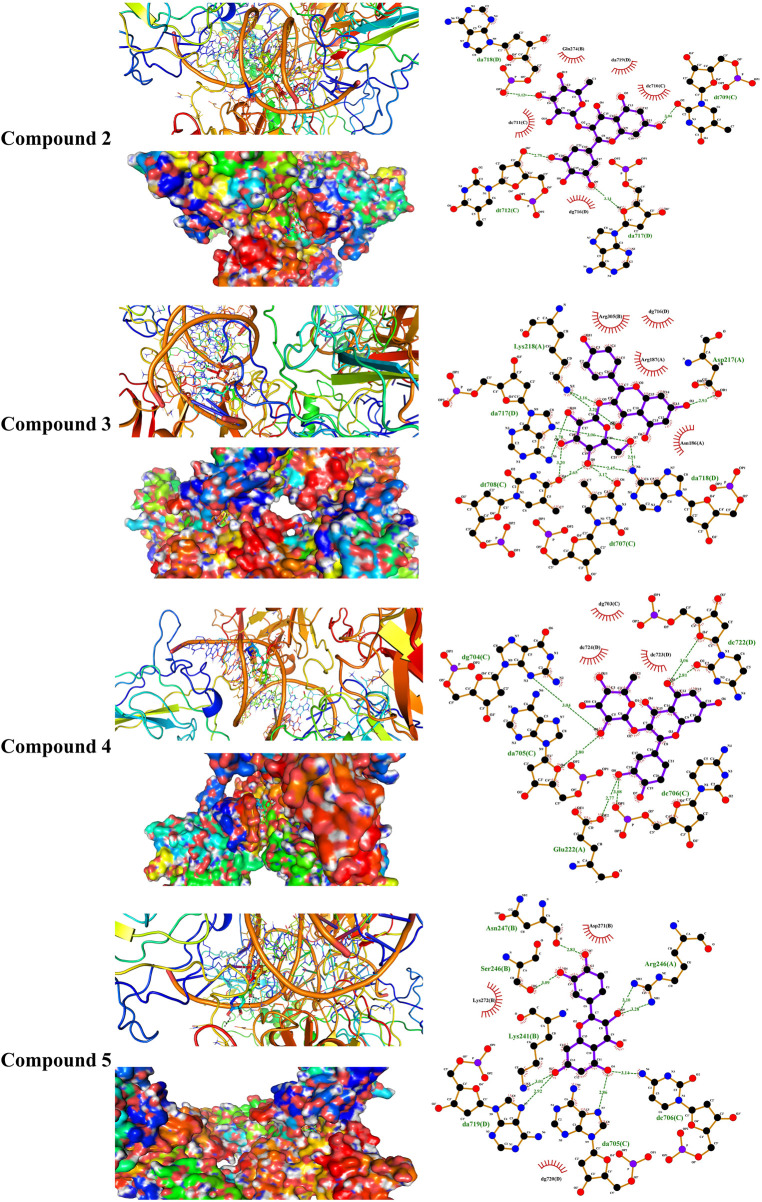
Molecular docking showing the binding modes of compounds **2**, **3**, **4**, and **5** with the NF-κB–DNA complex.

Liver NF-kB p65 and serum TNF-α and IL-1β were upregulated in diabetic rats as depicted in Figures 8A–C. Treatment with EPE noticeably decreased the assayed inflammatory markers in rats with diabetes. The binding affinity of the isolated flavonoids toward NF-kB was investigated with MD (Table 2; Figure 9 and Supplementary Figure S17). All compounds showed binding affinity marked by the polar bonding and hydrophobic interactions, and compounds **2**, **3**, **4**, and **5** showed the lowest binding energy (−9.5, −10.6, −9.8, and −9.6 kcal/mol, respectively).

**TABLE 2:** Binding affinities, interacting polar residues, and hydrophobic interactions of the compounds isolated from *E. peplus* with the NF-κB–DNA complex.

Compound	Binding energy (kcal/mol)	Polar bond	Hydrophobic interaction
**1**	−8.9	Gln274 and two DNA units	Five DNA units
**2**	−9.5	Four DNA units	Gln274 and four DNA units
**3**	−10.6	Asp217, Lys218, and four DNA units	Asn186, Arg187, Arg305, and one DNA unit
**4**	−9.8	Glu222 and four DNA units	Three DNA units
**5**	−9.6	Lys241, Ser246, Arg246, Asn247, and three DNA units	Asp271, Lys272, and one DNA unit
**6**	−9.1	Lys241, Asp271, Arg246, Asn247, and three DNA units	Ser246, Lys272, and one DNA unit
**7**	−6.4	Lys241 and three DNA units	Arg246, Lys272, and two DNA units

### 3.7 EPE upregulates adiponectin and PPARγ in diabetic rats

**FIGURE 10:**
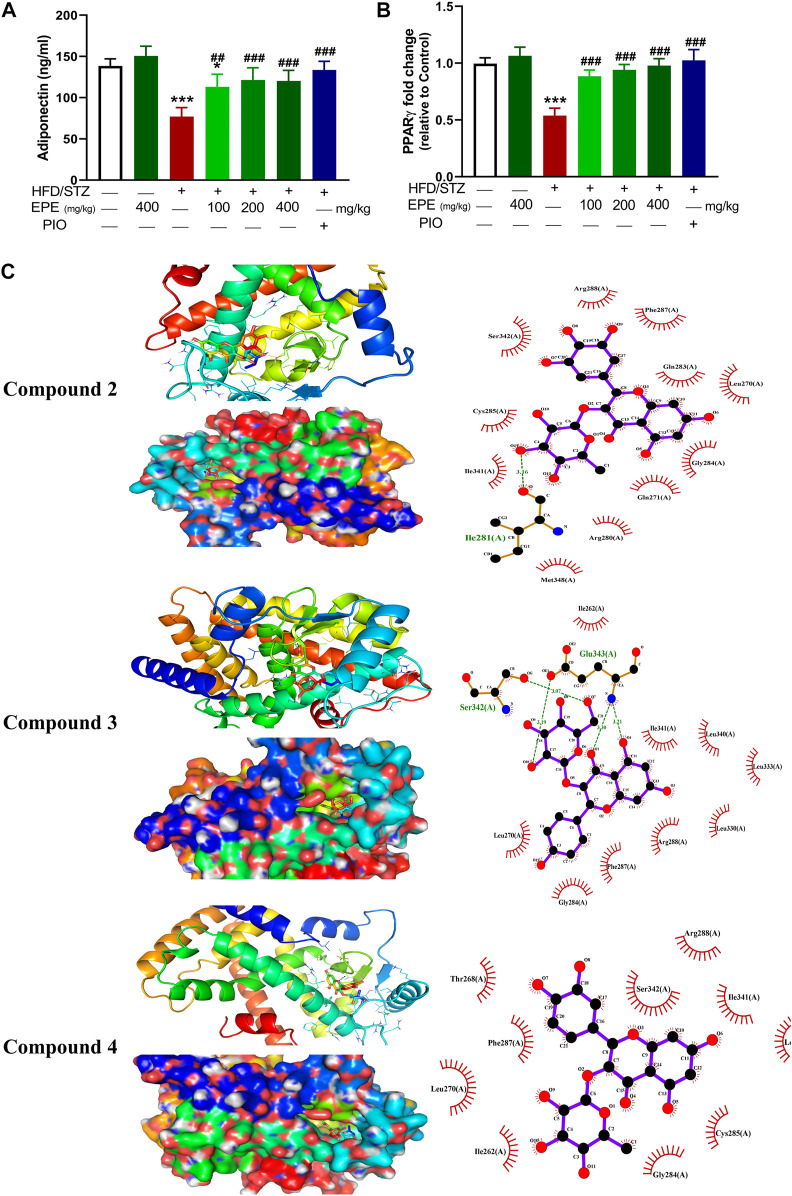
EPE increased serum adiponectin **(A)** and upregulated liver PPARγ mRNA **(B)** in diabetic rats. Data are mean ± SD (*n* = 6). *p < 0.05 and ***p < 0.001 vs. control. ##*p* < 0.01 and ###*p* < 0.001 vs. diabetic. **(C)** Molecular docking showing the binding modes of compounds **2**, **3**, and **4** with PPARγ.

Circulating adiponectin was declined in rats with diabetes, and all EPE doses effectively restored its levels (Figure 10A). The effect of EPE on PPARγ and the binding affinity of the isolated flavonoids were determined using qRT-PCR and MD, respectively. As shown in Figure 10B, diabetic rats exhibited significant downregulation of liver PPARγ, an effect that was reversed following treatment with all doses of EPE and the PPARγ agonist PIO. MD revealed the affinity of *E. peplus* flavonoids toward PPARγ, and compounds **3**, **4**, and **5** exhibited the lowest binding energy (−8.7, −8.0, and −8.0 kcal/mol, respectively) (Table 3; Figure 10C and Supplementary Figure S18I).

**TABLE 3:** Binding affinities, interacting polar residues, and hydrophobic interactions of the compounds isolated from *E. peplus* with PPARγ.

Compound	Binding energy (kcal/mol)	Polar bond	Hydrophobic interaction
**1**	−7.9	Gln271, Glu272, and Ser342	Ile262, Leu270, Gly284, Phe287, Arg288, Ile341, and Met348
**2**	−8.7	Ile281	Leu270, Gln271, Arg280, Gln283, Gly284, Cys285, Phe287, Arg288, Ile341, Ser342, and Met348
**3**	−8.0	Ser342 and Glu343	Ile262, Leu270, Gly284, Phe287, Arg288, Leu330, Leu333, Leu340, and Ile341
**4**	−8.0		Ile262, Thr268, Leu270, Gln271, Arg280, Gly284, Cys285, Phe287, Arg288, Ile341, and Ser342
**5**	−7.5	Glu259, Gln271, Arg280, and Glu291	Leu270, Gly284, Phe287, and Arg288
**6**	−7.4		Leu270, Gln271, Arg280, Gln283, Gly284, Phe287, Arg288, and Glu291
**7**	−5.8	Ser289, His323, and Met364	Cys285, Tyr327, Phe363, His449, Leu469, and Tyr473

## 4 Discussion

Plants of the genus *Euphorbia* showed a very promising anti-diabetic effect in STZ-, alloxan-, and sucrose-induced DM in rats (Kumar et al., 2010; Zafar et al., 2021; Mustafa et al., 2022), and the LD_50_ of most *Euphorbia* species was estimated to exceed 5,000 mg/kg (Abd-Elhakim et al., 2019). Herein, we explored the ameliorative effect of the flavonoid-rich fraction of *E. peplus* on hyperglycemia, IR, OS, and inflammation in HFD/STZ-induced T2D rats. The *in vitro* assays showed that EPE scavenged DPPH radicals in a concentration-dependent manner. Previous studies showed the DPPH radical-scavenging efficacy of plants of the genus *Euphorbia* such as *E. royleana* (Zafar et al., 2021). The DPPH assay data were supported by the ability of EPE to scavenge ABTS radicals, demonstrating its powerful RSA. ABTS assay is more reliable and accurate for the evaluation of RSA of phytoconstituents than DPPH (Floegel et al., 2011). The RSA of EPE could be directly related to the rich content of flavonoids that possess potent scavenging properties against free radicals (Kamel et al., 2016; Elsayed et al., 2020).

The effect of EPE on glucose intolerance and IR was investigated *in vivo* in rats with HFD/STZ-induced diabetes. HFD and STZ were employed to induce T2D as this model showed similarities to the disease in humans. Feeding a HFD results in IR, and STZ decreases insulin release by damaging *β*-cells (Breyer et al., 2005; Lee et al., 2011), leading to hyperglycemia. Together with IR, hyperglycemia is a characteristic feature of T2D and should be managed to prevent complications in different organs (Jellinger, 2007). Here, HFD/STZ-challenged animals showed hyperglycemia marked by glucose intolerance and IR. The developed T2D was consistent with our previous investigations, showing IR and hyperglycemia in HFD/STZ-induced rats (Mahmoud et al., 2012; Germoush et al., 2019; Elsayed et al., 2020; Abduh et al., 2023). The chronic hyperglycemia in this model was supported by the values of HbA1c%, a reliable marker for both diagnosis and prognosis of DM (American Diabetes Association, 2014) reported in our recent work (Abduh et al., 2023). Hyperglycemia was associated with hypoinsulinemia, and the development of IR as the value of HOMA-IR was revealed. Similar to these findings, elevated glucose, HbA1c%, and HOMA-IR along with decreased insulin were reported in HFD/STZ-challenged rats (Abduh et al., 2023). The declined insulin is due to damage caused to the pancreatic islets induced by STZ-mediated ROS generation and DNA damage (Lenzen, 2008). Although the early phase of damage is associated with increased insulin release as a compensatory mechanism, prolonged hyperglycemia and ROS release deteriorate the pancreatic islets and promote more *β*-cell damage and ultimately reduced insulin release (Ntimbane et al., 2016). The effects of excessive ROS include enhanced lipid peroxidation (LPO), massively increased cytosolic Ca^2+^, and diminished pancreatic antioxidants, effects that enhance the destruction of *β*-cells (Nahdi et al., 2017).

Treatment with EPE effectively ameliorated glucose intolerance and HOMA-IR, denoting its anti-hyperglycemic and insulin-sensitizing effects. These effects added support to the previously reported anti-hyperglycemic activity of plants of the same genus. For instance, *E. royleana* stem extract decreased fasting BG (FBG) and ameliorated glucose intolerance in diabetic rats (Zafar et al., 2021), and *E*. *helioscopia* alleviated BG and insulin in sucrose-fed rats (Mustafa et al., 2022). The ameliorative effect of EPE on hyperglycemia is a direct result of increased insulin secretion. Impaired insulin release and IR increase hepatic glucose output due to suppressed glycolysis and glycogenesis. Impaired insulin release and IR can also impair peripheral glucose uptake and hepatic gluconeogenesis, resulting in hyperglycemia (Nordlie et al., 1999). By alleviating insulin release and IR, EPE effectively ameliorated hyperglycemia possibly by modulating enzymes involved in glycogenesis and gluconeogenesis. This notion was supported by the findings of this study where EPE increased hexokinase and suppressed F-1,6-BPase, G-6-Pase, and glycogen phosphorylase, resulting in increased liver glycogen content. Hexokinase is involved in glucose oxidation and suppressed by IR and insulin deficiency. Suppressed hexokinase activity decreases glycolysis, and hence glucose accumulates in the blood (Gupta et al., 1999). Along with hexokinase suppression, insulin insufficiency activates G-6-Pase, F-1,6-BPase, and glycogen phosphorylase, resulting in enhanced gluconeogenesis and glycogenolysis (Roden and Bernroider, 2003). The improved insulin sensitivity and levels of EPE decreased glycogenolysis and gluconeogenesis and enhanced liver glycogen by modulating the activity of the involved enzymes. In addition to the determined enzymes, insulin activates glycogen synthase and suppresses glycogen phosphorylase (Postic et al., 2004), and this explains the alleviated glycogen levels following treatment with EPE. Owing to its role in glucose oxidation, the ameliorated FBG following EPE supplementation is a result of enhanced hexokinase activity. To further explore the effect of EPE on hexokinase activity, we carried out MD simulations of the binding affinity of the contained flavonoids toward the enzyme. All flavonoids revealed binding affinity marked by polar bonding toward important residues in the active site and dense hydrophobic interactions. Recent findings showed improvements in glycemic status and insulin sensitivity by plant extracts that modulate the carbohydrate-metabolizing enzymes (Germoush et al., 2019; Elsayed et al., 2020). In this context, Mustafa et al. (2022) related the anti-hyperglycemic effect of *E. helioscopia* in sucrose-fed rats to its ability to modulate the activities of pyruvate kinase, glucokinase, and phosphofructokinase.

In addition to hyperglycemia, dyslipidemia is found in T2D and can increase atherogenicity and the risk of cardiovascular disease (Reaven, 2005). Elevated serum lipids and decreased HDL-C in this study represent an atherogenic profile as previously described (Germoush et al., 2019). AIP, a marker of lipoprotein particle size that possesses a predictive value beyond that of the assayed lipids (Dobiásová, 2006), was increased in diabetic rats. The observed dyslipidemia is a direct result of IR and the enhanced lipolysis and decreased lipogenesis (Carpentier, 2021). Increased lipolysis provokes liver lipid accumulation which is also promoted by increased synthesis of free fatty acids (FFAs) that provoke lipogenesis within hepatocytes (Mohamed et al., 2016). Lipid accumulation in hepatocytes causes cell injury, thereby aggravating IR, hyperglycemia, and dyslipidemia (Levinthal and Tavill, 1999). Herein, lipids were increased in the liver, and circulating transaminases were elevated in diabetic rats as previously reported (Elsayed et al., 2020; Abduh et al., 2023). EPE effectively ameliorated serum and liver lipids, effects that were directly related to the enhanced insulin release and sensitivity.

Owing to the involvement of OS and inflammation in provoking IR and the complications of DM (Mahmoud et al., 2012; Mahmoud, 2017), we explored the ability of EPE to suppress these pathological processes. Diabetic rats showed OS and inflammatory reactions marked by elevated MDA, NO, NF-kB, TNF-α, and IL-1β and declined antioxidants. OS, defined by excess ROS and decreased antioxidants, is a key mechanism in IR and can damage cells and alter multiple signaling pathways. Hyperglycemia can increase the production of ROS and lead to OS by activating NADPH oxidases and promoting mitochondrial dysfunction (Jimenez et al., 2018). Excess ROS can activate pathways related to increased pro-inflammatory cytokines, and both can impair insulin signaling, leading to IR and glucose accumulation in the blood (Rösen et al., 2001). The altered insulin levels shift the signaling where PI3K phosphorylates Rac, resulting in increased NADPH oxidase 4-mediated ROS generation (Campa et al., 2015). Excess ROS activates casein kinase-2 followed by retromer that alters glucose transporter-4 membrane translocation and impair glucose uptake (Ma et al., 2014). ROS can also increase mitochondrial fission that stimulates stress responses and impairs insulin signaling and has been linked to IR as well as apoptosis (Jheng et al., 2012). Pro-inflammatory cytokines trigger IR by altering insulin signaling and many kinases. The elevated IL-1β and TNF-α reported in this study can impair insulin-stimulated uptake of glucose, stimulate lipolysis and gluconeogenesis, and inhibit tyrosine phosphorylation of insulin receptor substrate-1 and protein kinase B activation (Green et al., 1994; Del Aguila et al., 1999; Jager et al., 2007). Therefore, attenuation of OS and pro-inflammatory cytokines can attenuate IR and increase insulin signaling, activity, and stimulated glucose uptake.

EPE enhanced antioxidants and prevented OS and inflammation in diabetic rats in this investigation. In addition to its *in vitro* RSA, EPE prevented LPO, enhanced antioxidants, and suppressed NF-kB and cytokines in diabetic rats. The suppression of inflammation following EPE supplementation was supported by *in silico* investigations that showed the ability of flavonoids to bind strongly with NF-kB through multiple polar bonding and hydrophobic interactions. The attenuation of these pathological processes contributed to the anti-hyperglycemic and insulin-sensitizing effects of EPE. Numerous studies showed the beneficial effects of antioxidants and plant extracts that are rich in antioxidant phytochemicals against hyperglycemia and IR (Mahmoud, 2013; Mahmoud et al., 2017; Germoush et al., 2019). The antioxidant and anti-inflammatory role of EPE is related to its content of flavonoids which possess potent RSA and showed benefits against DM (Mahmoud, 2013; Mahmoud et al., 2017; Germoush et al., 2019; Abukhalil et al., 2021). In diabetic patients, the supplementation of flavonoids improved glycemic and lipidemic statuses and antioxidants and decreased inflammatory markers (Li et al., 2015). In obese patients, the consumption of flavonoids positively affected the metabolic status by lowering systemic oxidation and enhancing insulin sensitivity (Suliburska et al., 2012).

The beneficial effects of EPE could also be linked to the upregulation of adiponectin and PPARγ. EPE increased serum adiponectin that participated, at least in part, in the amelioration of hyperglycemia. Adiponectin exerts insulin-sensitizing effects and possesses anti-inflammatory activity, and experimental evidence revealed that it ameliorated hyperglycemia in HFD-fed rodents (Fruebis et al., 2001; Yamauchi et al., 2001). Despite its ameliorated hyperglycemia in T1D and T2D in rodents, high adiponectin doses didn’t affect BG in normal animals. These findings suggested that the downregulation of glycogenolysis and gluconeogenesis mediated its anti-hyperglycemic effects. Accordingly, adiponectin decreased glucose production in rat hepatocytes and G-6-Pase mRNA abundance in mice (Berg et al., 2001; Combs et al., 2001). It can also upregulate liver CD36, PPARα, and UCP-2, effects that were related to the increase in insulin sensitivity (Yamauchi et al., 2001). EPE upregulated liver PPARγ, and its flavonoids were shown to dock into the PPARγ active site through polar bonding and hydrophobic interactions. The activation of PPARγ is a key mechanism for ameliorating hyperglycemia, and IR and PPARγ agonists, such as PIO, increase insulin sensitivity and ameliorate hyperglycemia, dyslipidemia, OS, and inflammation (Tontonoz and Spiegelman, 2008). PPARγ suppresses OS and inflammation by enhancing antioxidant enzymes (Okuno et al., 2010), inhibiting the activation of NF-κB both directly and indirectly (Kersten et al., 2000; Remels et al., 2009), and preventing ROS generation from NADPH oxidases (Hwang et al., 2005). However, the lack of PPARγ protein expression data could be considered a limitation to this study.

## 5 Conclusion

This investigation introduces new information that *E. peplus* is rich in flavonoids and possesses potent radical-scavenging and anti-diabetic efficacies. EPE ameliorated hyperglycemia, IR, OS, dyslipidemia, and inflammation in rats with T2D. In addition, EPE modulated carbohydrate-metabolizing enzymes and enhanced antioxidants, adiponectin, and PPARγ. *In silico* findings revealed the binding affinity of *E. peplus* constituents toward hexokinase, NF-kB, and PPARγ. Therefore, *E. peplus* could be a promising candidate for the development of a potent anti-hyperglycemic and insulin-sensitizing agent. However, further investigations to determine other molecular mechanism(s) of action are needed.

## Data Availability

The original contributions presented in the study are included in the article/Supplementary Material; further inquiries can be directed to the corresponding author.
