# Supplementary material for: A flavonoid-rich fraction of Euphorbia peplus attenuates hyperglycemia, insulin resistance, and oxidative stress in a type 2 diabetes rat model
Source: Front Pharmacol. 2023 Jun 16;14:1204641. doi: 10.3389/fphar.2023.1204641 (PMC10311489; doi:10.3389/fphar.2023.1204641)
Supplement: Supplementary file 1 [file DataSheet1.docx]

Supplementary Material

Title:

A flavonoid-rich fraction of *Euphorbia peplus* attenuates hyperglycemia, insulin resistance and oxidative stress in a rat model of type 2 diabetes

Running title: Anti-diabetic activity of *Euphorbia peplus* flavonoids.

Authors & Affiliations:

Reem S. Alruhaimi^1^, Gomaa Mostafa-Hedeab^2,3^, Maisa Siddiq Abduh^4,5^, Albandari Bin-Ammar^6^, Emad H. M. Hassanein^7^, Emadeldin M. Kamel^8^, Ayman M. Mahmoud^9,10^*

^1^Department of Biology, College of Science, Princess Nourah bint Abdulrahman University, Riyadh 11671, Saudi Arabia.

^2^Pharmacology Department, Medical College, Jouf University, Sakaka 72388, Saudi Arabia.

^3^Pharmacology Department, Faculty of Medicine, Beni-Suef University, Beni-Suef 62514, Egypt.

^4^Immune Responses in Different Diseases Research Group, Department of Medical Laboratory Sciences, Faculty of Applied Medical Sciences, King Abdulaziz University, Jeddah 21589, Saudi Arabia.

^5^Center of Excellence in Genomic Medicine Research, King Abdulaziz University, Jeddah 22252, Saudi Arabia.

^6^Department of Clinical Nutrition, College of Applied Medical Sciences, University of Hail, Saudi Arabia.

^7^Department of Pharmacology and Toxicology, Faculty of Pharmacy, Al-Azhar University, Assiut 71524, Egypt.

^8^Chemistry Department, Faculty of Science, Beni-Suef University, Beni-Suef 62514, Egypt.

^9^Department of Life Sciences, Faculty of Science and Engineering, Manchester Metropolitan University, Manchester M1 5GD, UK

^10^Physiology Division, Zoology Department, Faculty of Science, Beni-Suef University, Beni-Suef 62514, Egypt

# * Correspondence:

# Ayman M. Mahmoud

# [ayman.mahmoud@science.bsu.edu.eg](mailto:ayman.mahmoud@science.bsu.edu.eg) & a.mahmoud@mmu.ac.uk

**Supplementary Methods:**

***In-silico* molecular docking analysis**

Molecular docking analysis was employed to explore the binding profile of *E. peplus* isolated flavonoids (**1**-**7**) with three target proteins namely, PPARγ (PDB ID: 2PRG), NF-κB-DNA complex (PDB ID: 1LE9) and hexokinase II (PDB ID: 2NZT). DFT calculations performed in this work were executed using Gaussian 16 software package (Frisch et al., 2016). The geometrical structures of isolated flavonoids were fully optimized at the B3LYP level of theory (Becke, 1988;Lee et al., 1988;Becke, 1993) using the basis set 6-311G + (d, p) (Hehre et al., 1986). The pdb three-dimensional crystal structures of target proteins employed in this study were obtained from the Protein Data Bank (RCSB PDB), meanwhile the pdb structures of isolated flavonoids were constructed using UCSF Chimera 1.16 software (Pettersen et al., 2004). Also, UCSF Chimera was utilized for the initial optimization of various targets, these optimization processes include removal of native ligand and stripping out water molecules and nonstandard residues. Molecular docking investigation in this work was carried out using AutoDock Vina and Autodock Tools (ADT) v1.5.6 software package from the Scripps Research Institute (Trott and Olson, 2010). The structures of the targets and ligands must be appropriately optimized prior to the docking process. Initially, ADT was employed to prepared various proteins by removing water molecules, adding polar hydrogens and identifying the binding site of different targets by using the native ligand through adjusting the grid box to the most suitable active site (Kamel and Lamsabhi, 2020;2021). The software PyMOL v2.4 was used to generate high resolution images and to examine binding sites, whereas UCSF Chimera 1.16 was used for molecular visualization.

Supplementary Table I. Primers used for qRT-PCR.

| Gene | GenBank accession number | Primer sequence (5′-3′) | Amplicon size  (bp) |
| --- | --- | --- | --- |
| PPARγ | NM_001145367.1 | F: GGACGCTGAAGAAGAGACCTG  R: CCGGGTCCTGTCTGAGTATG | 135 |
| GAPDH | NM_017008.4 | F: GCATCTTCTTGTGCAGTGCC  R: GGTAACCAGGCGTCCGATAC | 91 |


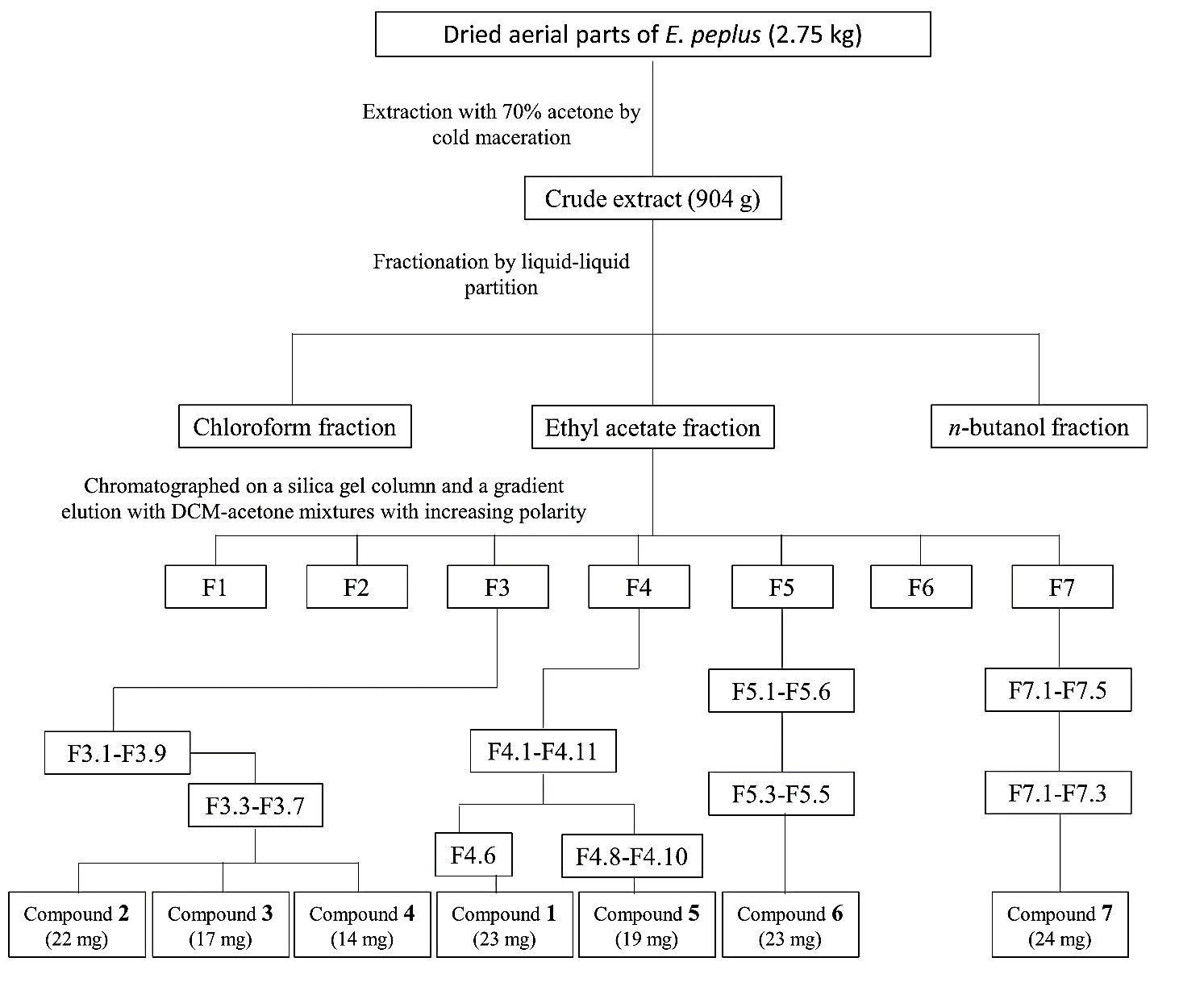


Fig. S1. Flowchart for the extraction and isolation of secondary metabolites from *E. peplus*.

***Isoquercetin (1):*** yellow amorphous powder, ^1^H NMR (500 MHz, CD_3_OD) δ_H_: 7.73 (1H, d, *J* = 2.2 Hz, H-2 ʹ), 7.61 (1H, dd, *J* = 8.5, 2.2 Hz, H-6 ʹ), 6.88 (1H, d, *J* = 8.5 Hz, H-5 ʹ), 6.41 (1H, d, *J* = 2.0 Hz, H-8), 6.21 (1H, d, *J* = 2.0 Hz, H-6), 5.26 (1H, d, *J* = 7.7, H-1ʹʹ) and 3.22-3.74 (5H, m, H-2ʹʹ-H-6ʹʹ) ^13^C NMR (125 MHz, CD_3_OD) δ_C_: 158.93 (C-2), 135.59 (C-3), 179.42 (C-4), 105.52 (C-10), 163.04 (C-5), 100.08 (C-6), 166.65 (C-7), 94.83 (C-8), 158.54 (C-9), 123.07 (C-1ʹ), 117.55 (C-2ʹ), 145.93 (C-3ʹ), 149.88 (C-4ʹ), 114.91 (C-5ʹ), 123.17 (C-6ʹ), 104.34 (C-1ʹʹ), 75.73 (C-2ʹʹ), 78.13 (C-3ʹʹ), 71.23 (C-4ʹʹ), 78.40 (C-5ʹʹ), 62.55 (C-6ʹʹ).


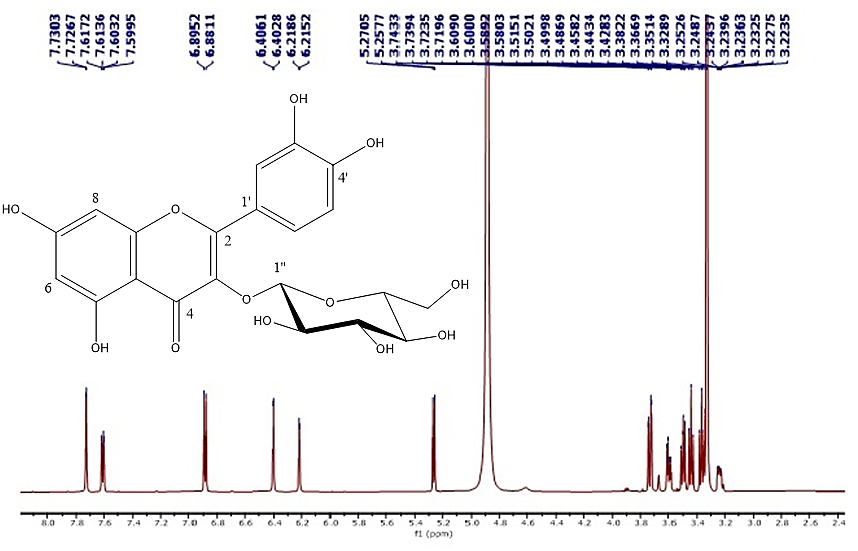


Fig S2. ^1^H NMR Spectrum of compound **1** (CD_3_OD; 500 MHz).


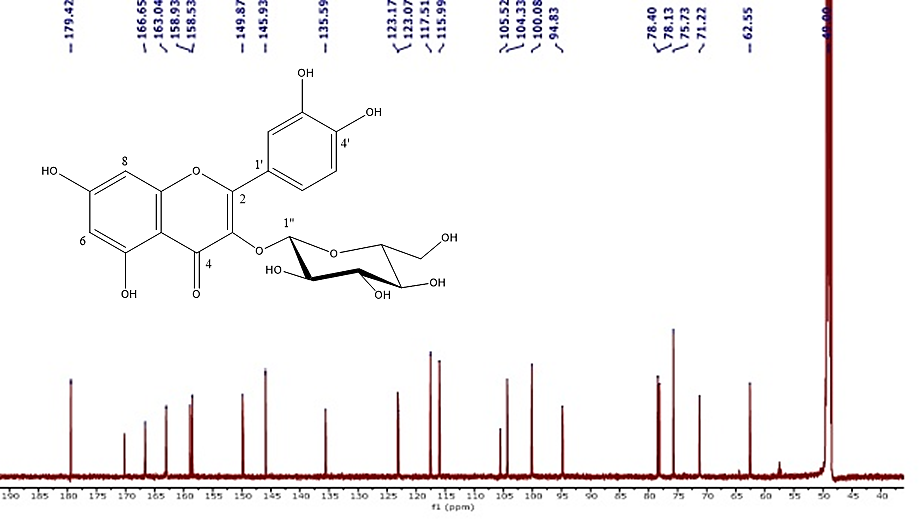


Fig S3. ^13^C NMR Spectrum of compound **1** (CD_3_OD; 125 MHz)

***Myricitrin (2):*** yellow amorphous powder, ^1^H NMR (500 MHz, CD_3_OD) δ_H_: 6.91 (1H, *s*, H-2’ and H-6’), 6.38 (1H, *d*, *J*=2.4 Hz, H-8), 6.21 (1H, *d*, *J* = 2.4 Hz, H-6), 5.23 (1H, *d*, *J* = 1.7 Hz, H-1’’), 3.57-4.01 (4H, *m*, H-2”-H-5”), 0.86 (3H, *d*, *J* = 5.9 Hz, H-6’’). ^13^C NMR (125 MHz, CD3OD) δ_C_: 178.25 (C-4), 164.67 (C-7), 161.78 (C-5), 157.96 (C-2), 156.88 (C-9), 146.24 (C-3’ and C-5’), 136.93 (C-4’), 134.75 (C-3), 120.09 (C-1’), 108.38 (C-2’ and C-6’), 104.50 (C-10), 102.40 (C-1’’), 99.14 (C-6), 94.00 (C-8), 71.74 (C-4’’), 71.02 (C-3’’), 70.86 (C-5’’), 70.48 (C-2’’), 17.99 (C-6’’).


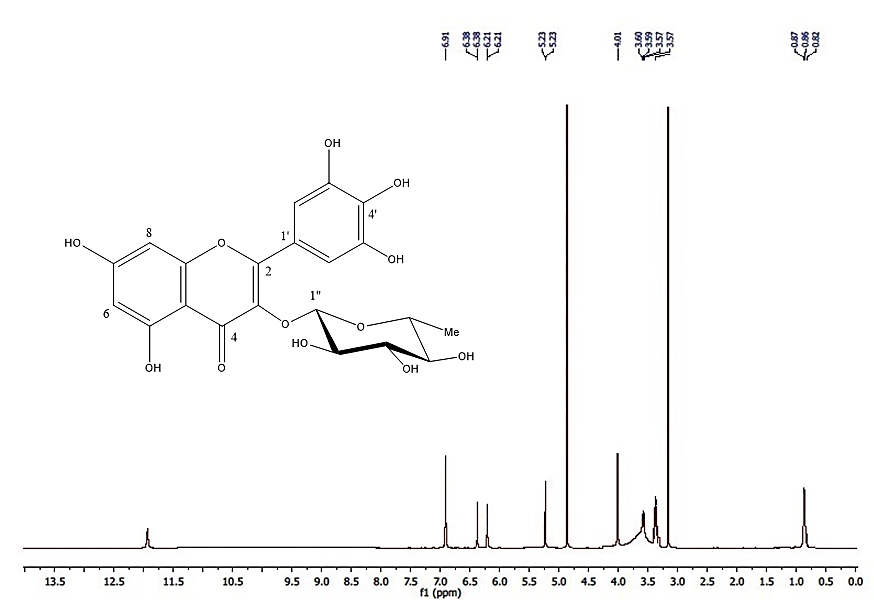


Fig S4. ^1^H NMR Spectrum of compound **2** (CD_3_OD; 500 MHz).


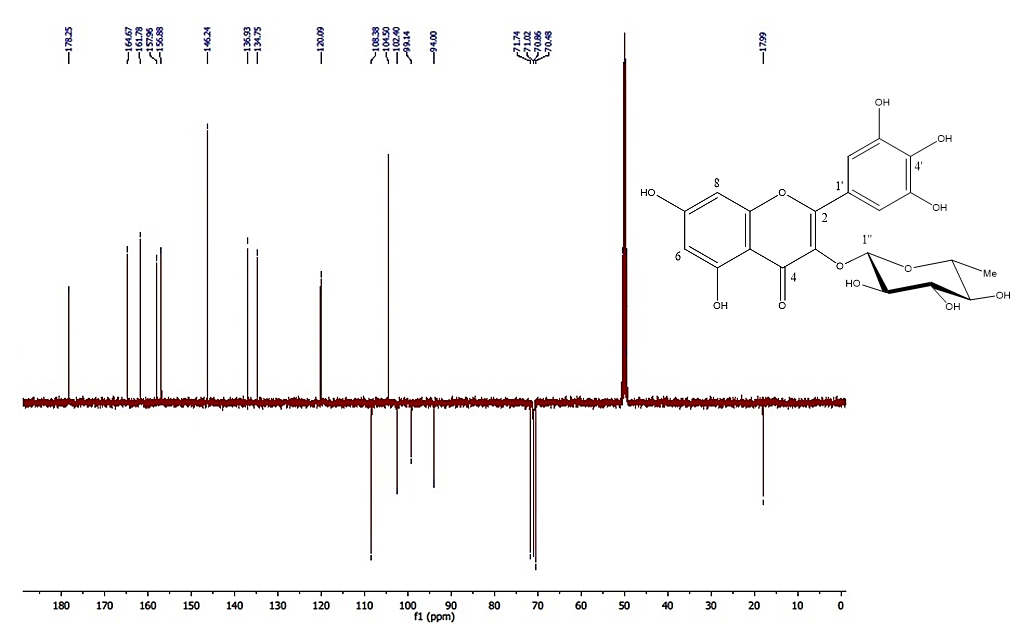


Fig S5. ^13^C NMR Spectrum of compound **2** (CD_3_OD; 125 MHz).

***Astragalin (3)****:* yellow amorphous powder, ^1^H NMR (500 MHz, CD_3_OD) δ_H_: 8.12 (1H, d, *J* = 9.0 Hz, H-2ʹ, 6ʹ), 6.9 (1H, d, *J* = 9.0 Hz, H-3ʹ, 5ʹ), 6.75 (1H, d, *J* = 1.95 Hz, H-8), 6.44 (1H, d, *J* = 2.0 Hz, H-6), 5.45 (1H, d, *J* = 7.45 Hz, glc-H-1ʹʹ) and 3.44-4.04 ((5H, m, H-2''- H-6''); ^13^C NMR (125 MHz, CD_3_OD) δ_c_: 177.5 (C-4), 162.9 (C-7), 160.8 (C-5), 157.7 (C-4ʹ), 148.8 (C-9), 137.7 (C-2), 131.5 (C-2ʹ, 6ʹ) 123.5 (C-3), 116.7 (C-1ʹ), 112.4 (C-3ʹ, 5ʹ), 106.4 (C-10), 99.8 (glc-C-1ʹʹ), 99.15 (C-6), 97.0 (C-8), 73.5 (glc-C-3ʹʹ), 73.2 (glc-C-5ʹʹ), 71.2 (glc-C-2ʹʹ), 70.4 (glc-C-4ʹʹ) and 62.2 (glc-C-6ʹʹ).


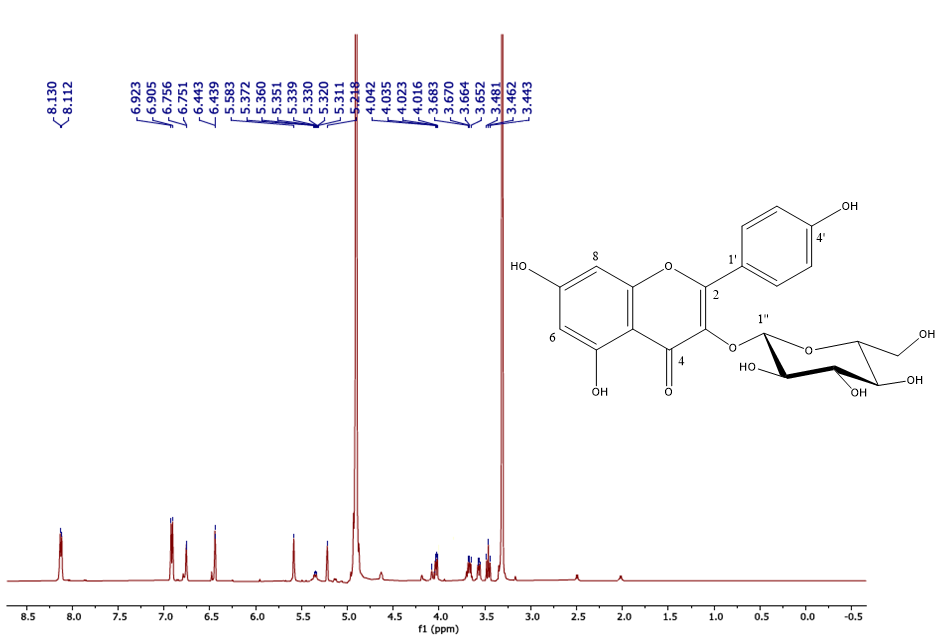


Fig S6. ^1^H NMR Spectrum of compound **3** (CD_3_OD; 500 MHz).


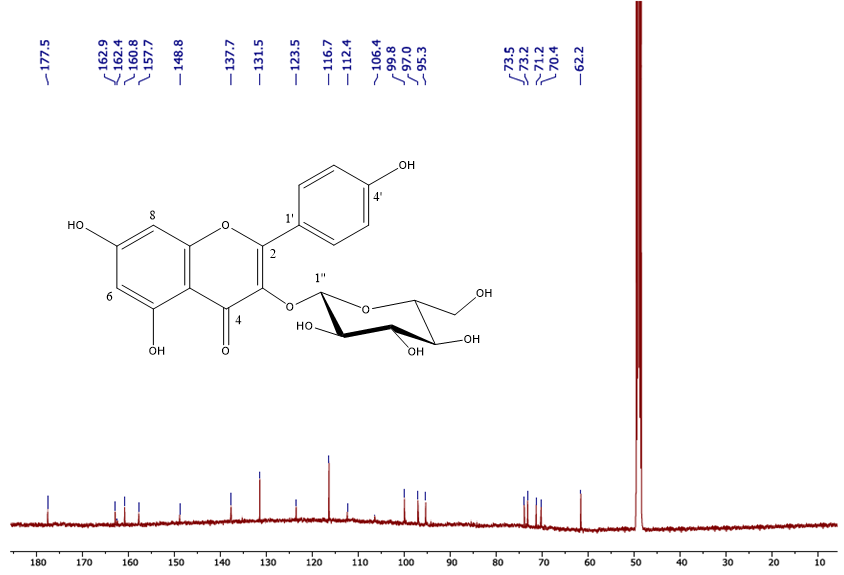


Fig S7. ^13^C NMR Spectrum of compound **3** (CD_3_OD; 125 MHz).

***Quercitrin (4)****:* yellow amorphous powder, ^1^H NMR (500 MHz, CD_3_OD) δ_H_: 7.30 (1H, d*, J* = 2.4 Hz, H-2' ), 7.26 (1H, dd*, J* = 2.4 Hz, and *J* = 8.6Hz, H-6' ), 6.89 (1H, d*, J* = 8.6 Hz, H-5'), 6.40 (1H, d, *J* = 2.3 Hz, H-8), 6.21 (1H, d, *J* = 2.3 Hz, H-6), 5.26 (1H, d, *J* = 1.52 Hz, H-1''), 3.15-3.98 (4H, m, H-2''- H-5'') and 0.83 (3H *,*d, *J*  = 5.71 Hz, H-6''); ^13^C NMR (125 MHz, CD_3_OD) δ_c_: 178.19 (C-4), 164.80 (C-7), 161.75 (C-5),157.75 (C-2), 156.92 (C-9), 148.93 (C-4'), 145.68 (C-3'), 134.67 (C-3), 121.58 (C-1'), 121.19 (C-6'), 116.12 (C-5'), 115.94 (C-2'), 104.50 (C-10), 102.29 (C-1''), 99.20 (C-6), 94.12 (C-8), 71.65 (C-4''),71.05 (C-3''), 70.82 (C-2''), 70.52 (C-5'') and 17.96 (C-6'').

***
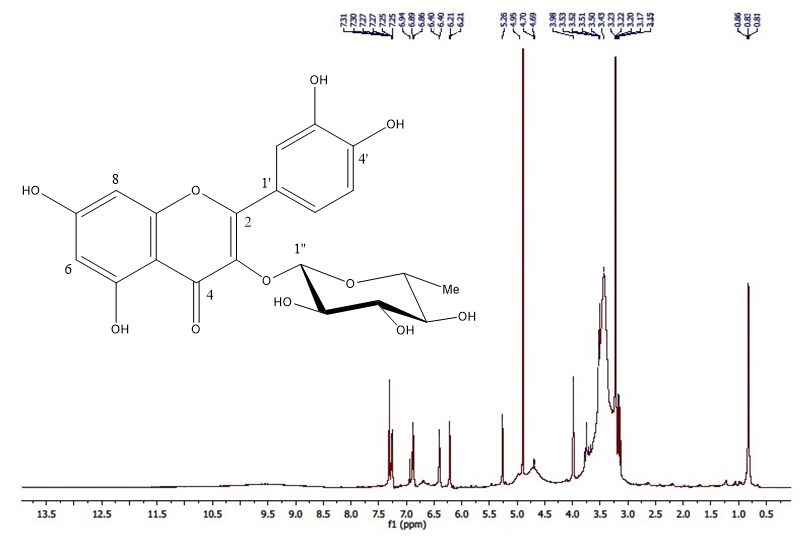
***

Fig S8. ^1^H NMR Spectrum of compound **4** (CD_3_OD; 500 MHz).


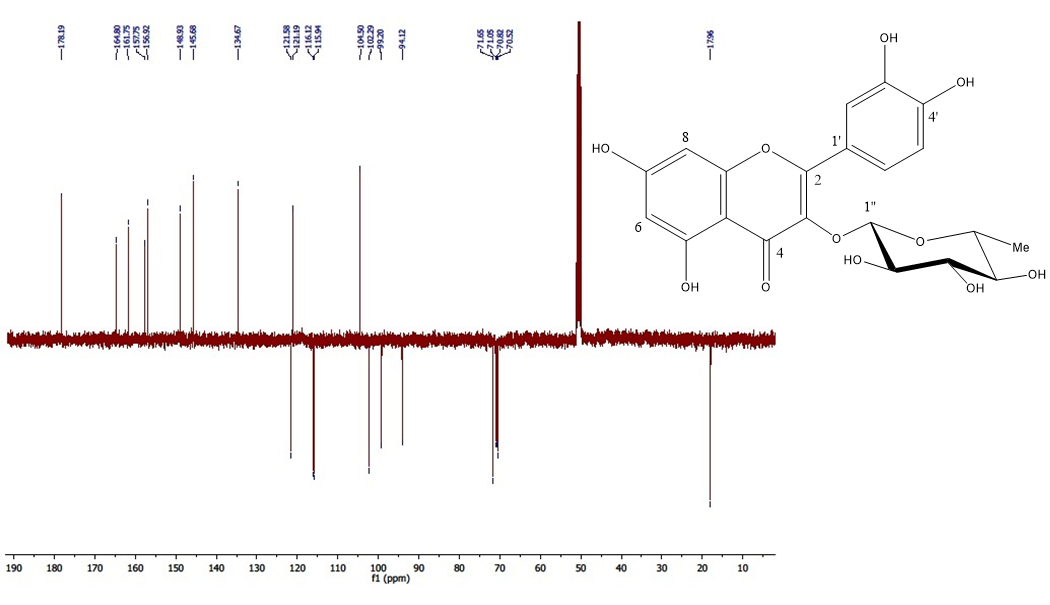


Fig S9. ^13^C NMR Spectrum of compound **4** (CD_3_OD; 125 MHz).

***Quercetin (5)*:** yellow amorphous powder, ^1^H NMR (500 MHz, CD_3_OD) δ_H_: 7.68 (1H, d, J = 2.1 Hz, H-2' ), 7.56 (1H, dd , J = 2.1 Hz, and 8.4 Hz, H-6' ), 6.91 (1H, d, J = 8.4 Hz, H-5'), 6.45 (1H, d, J = 1.8 Hz, H-8) and 6.21 (1H, d, J = 1.8 Hz, H-6); ^13^C NMR (125 MHz, CD_3_OD) δ_c_:147.24 (C-2), 136.21 (C-3), 176.30 (C-4), 161.14 (C-5), 98.69 (C-6), 164.48 (C-7), 93.84 (C-8), 156.83 (C-9), 103.42 (C-10), 122.42 (C-1'), 116.11 (C-2'), 145.54 (C-3'), 148.17 (C-4'), 115.50 (C-5') and 120.45 (C-6').


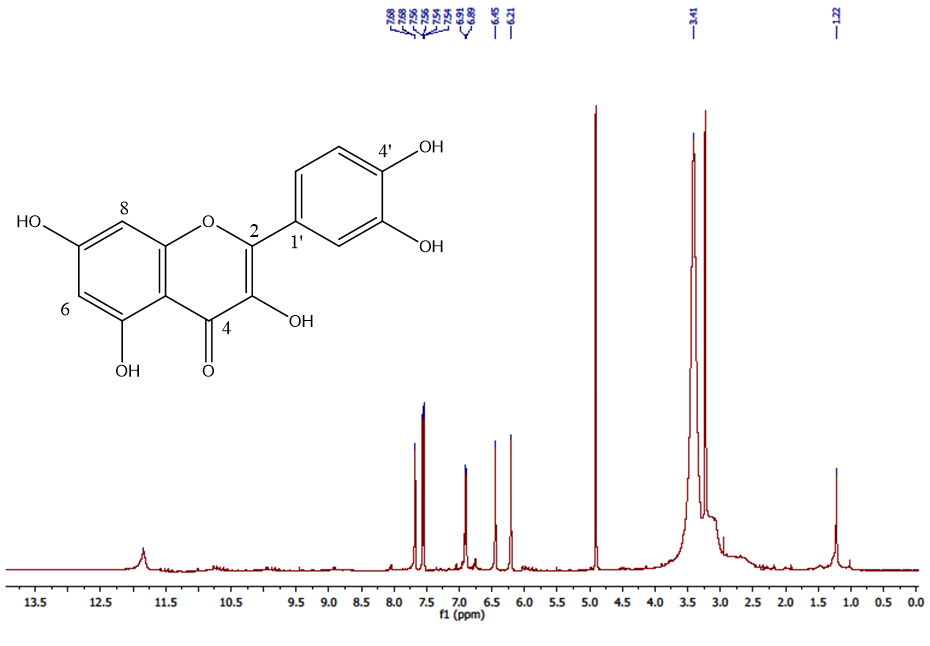


Fig S10. ^1^H NMR Spectrum of compound **5** (CD_3_OD; 500 MHz).


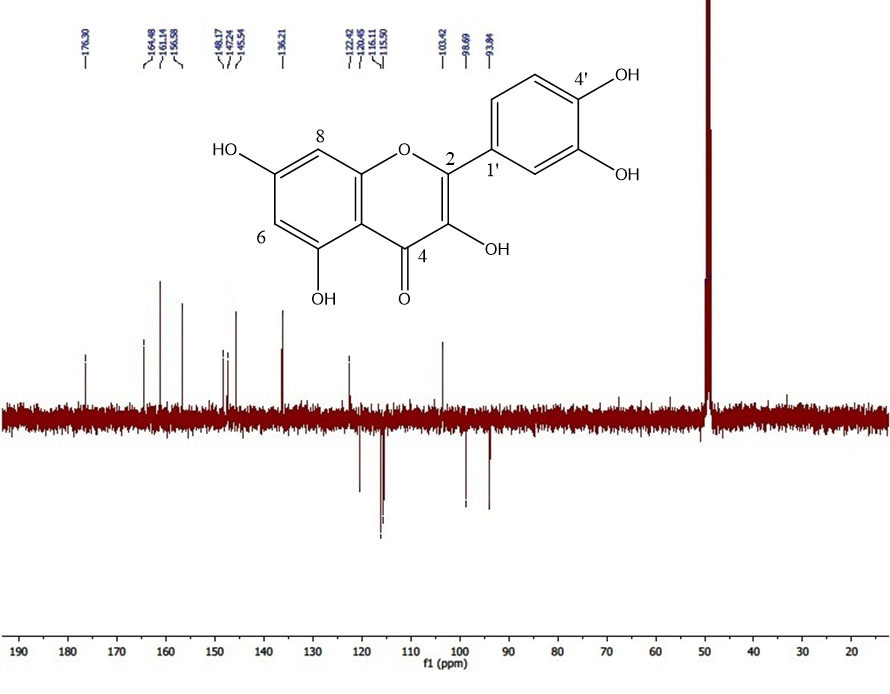


Fig S11. ^13^C NMR Spectrum of compound **5** (CD_3_OD; 125 MHz).

***Kaempferol (6):*** yellow crystalline solid ^1^H NMR (500 MHz, CD3OD) δ_H_: 7.69 (2H, d*, J* = 8.5 Hz, H-2', H-6'), 6.89 (2H, d*, J* = 8.5 Hz, H-3', H-5'), 6.38 (1H, d, *J* = 2.0 Hz, H-8) and 6.19 (1H, d, *J* = 2.0 Hz, H-6); ^13^C NMR (125 MHz, CD_3_OD) δ_c_: 176.04 (C-4), 162.91 (C-5), 161.19 (C-4′), 161.03 (C-7), 157.48 (C-9), 156.54 (C-2), 141.90 (C-3), 136.16 (C-2′, C-6′), 121.5 (C-1′), 115.43 (C-3′, C-5′), 103.34 (C-10), 98.70 (C-6) and 93.67 (C-8).


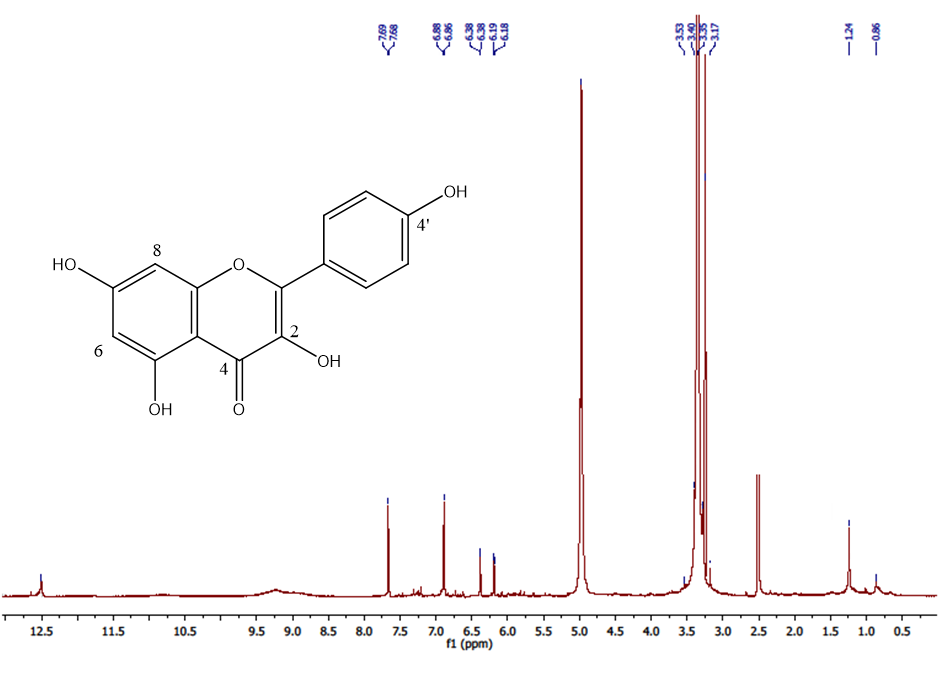


Fig S12. ^1^H NMR Spectrum of compound **6** (CD_3_OD; 500 MHz).


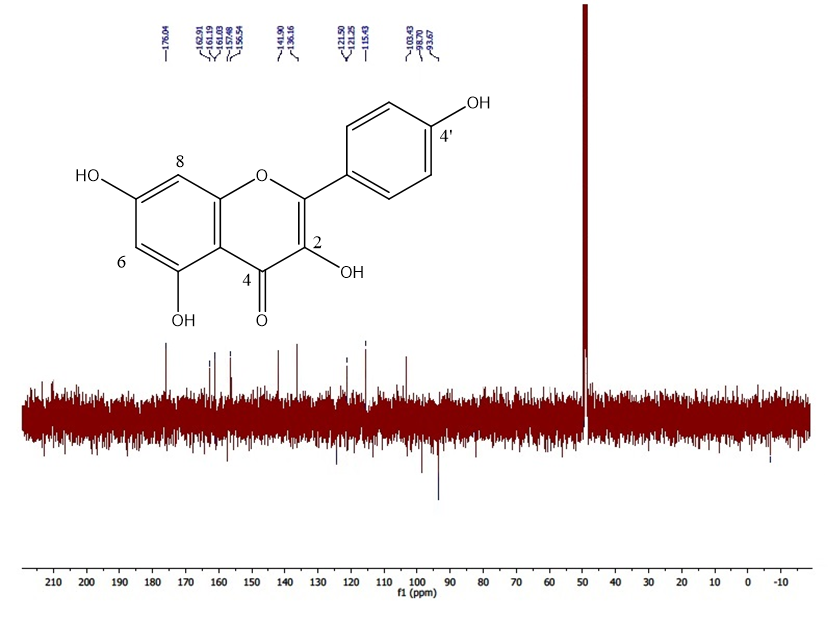


Fig S13. ^13^C NMR Spectrum of compound **6** (CD_3_OD; 125 MHz).

***Methyl gallate (7)*:** yellowish-white powder, ^1^H NMR (500 MHz, CD3OD) δ_H_: 6.94 (2H, s*,* H-2, H-6) and 3.74 (3H, s, OMe); ^13^C NMR (125 MHz, CD_3_OD) δ_c_: 166.81 (C-7), 146.05 (C-3, C-5), 138.92 (C-4), 119.74 (C-1), 108.97 (C-2, C-6) and 52.05 (OMe).


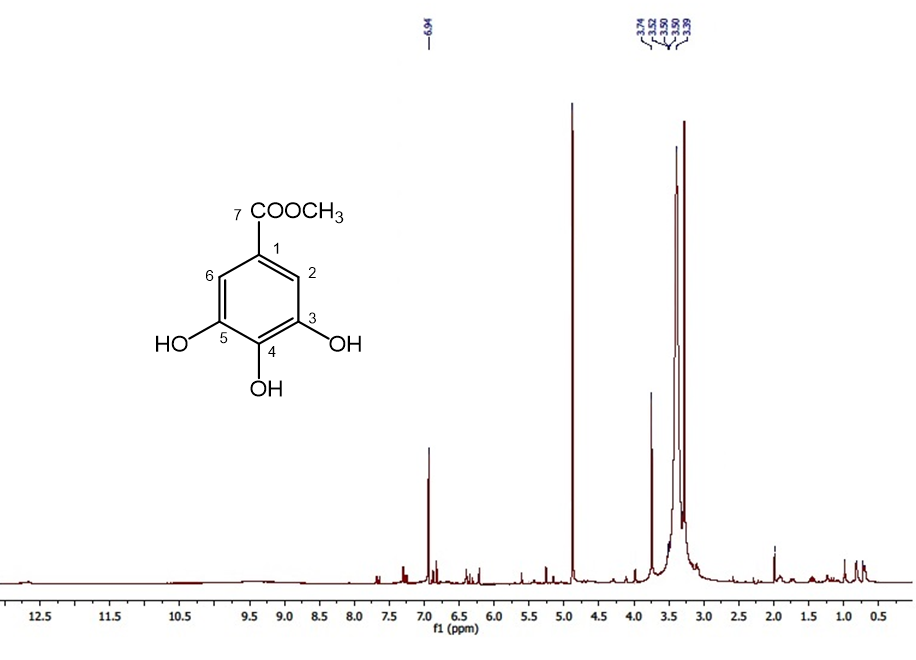


Fig S14. ^1^H NMR Spectrum of compound **7** (CD_3_OD; 500 MHz).


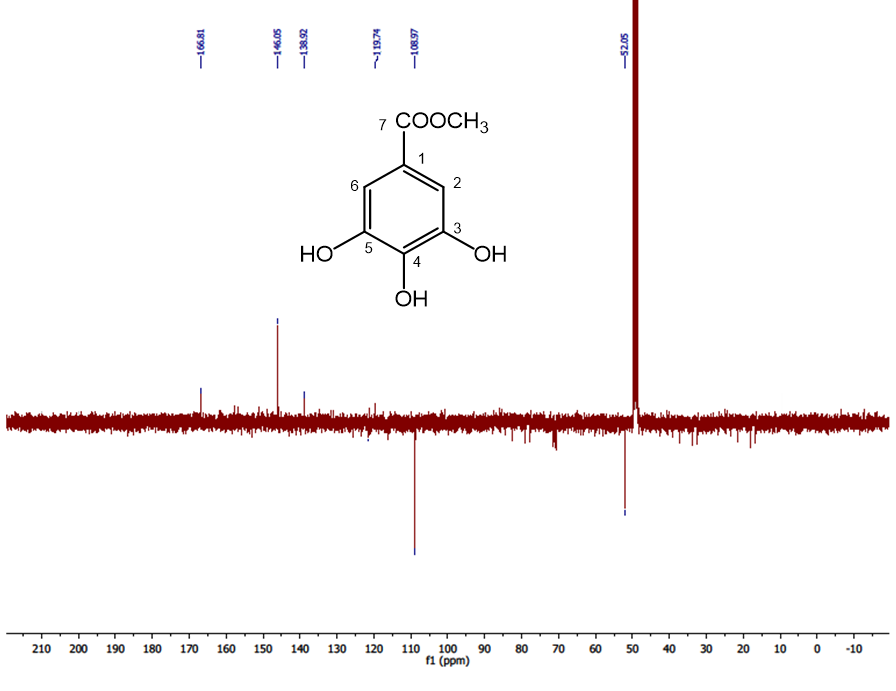


Fig S15. ^13^C NMR Spectrum of compound **7** (CD_3_OD; 125 MHz).


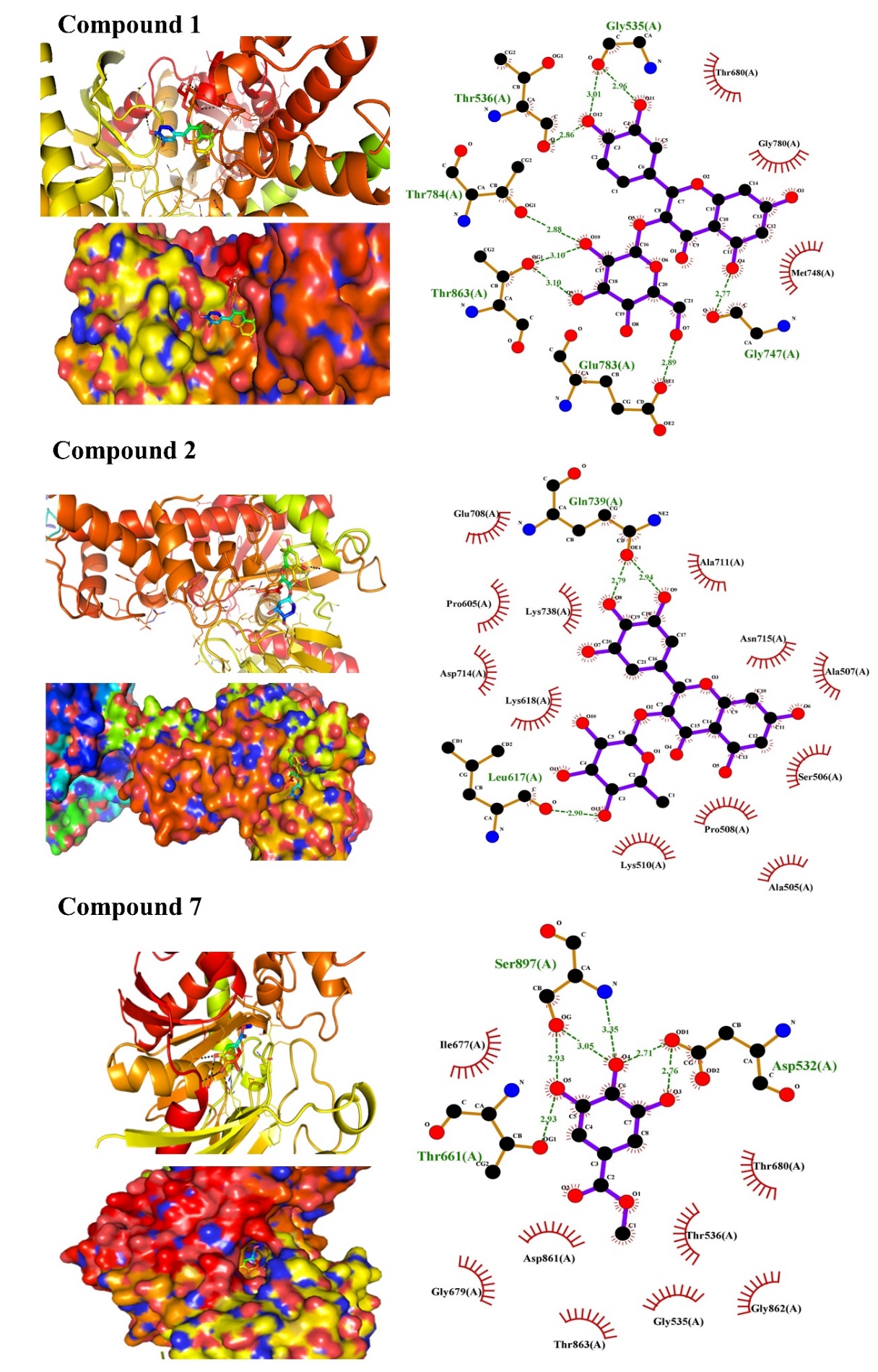


Supplementary Fig. S16. Molecular docking showing the binding modes of compounds **1**, **2** and **7** with hexokinase.


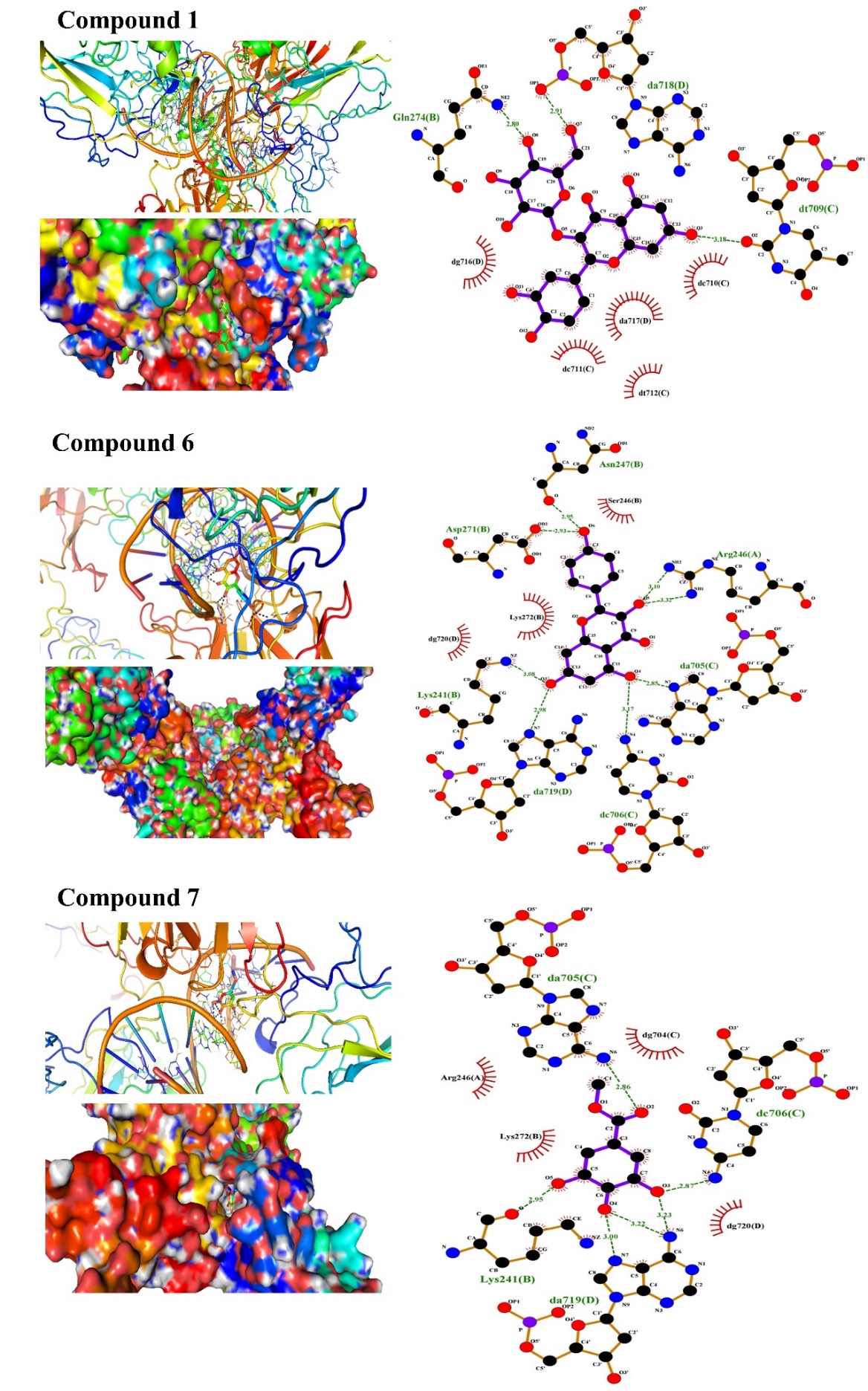


Supplementary Fig. 17. Molecular docking showing the binding modes of compounds **1**, **6** and **7** with NF-κB-DNA complex.


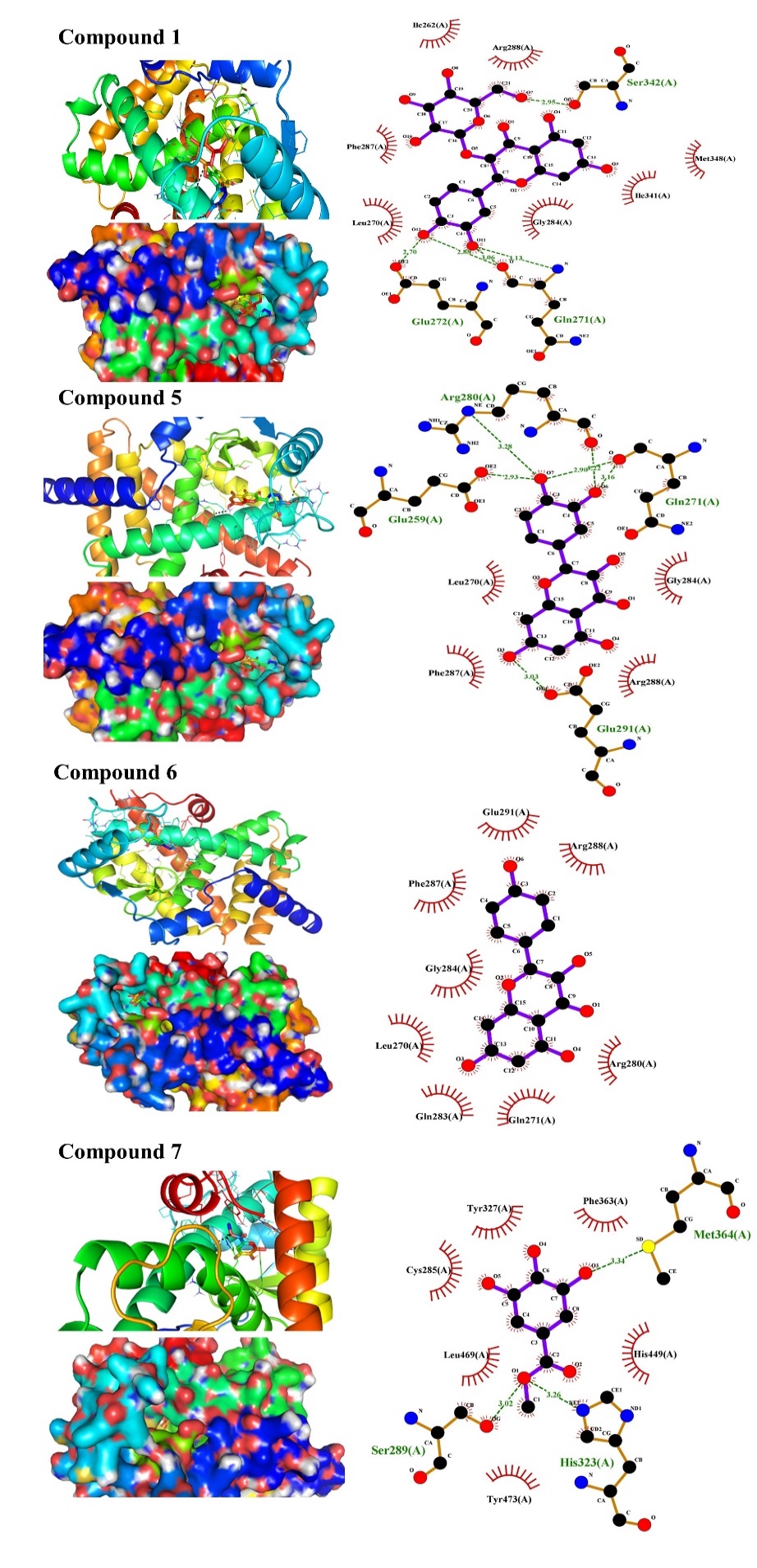


Supplementary Fig. 18. Molecular docking showing the binding modes of compounds **1**, **5, 6** and **7** with PPARγ.

**References:**

Becke, A.D. (1988). Density-functional exchange-energy approximation with correct asymptotic behavior. *Physical review A* 38**,** 3098.

Becke, A.D. (1993). Density‐functional thermochemistry. III. The role of exact exchange. *The Journal of Chemical Physics* 98**,** 5648-5652.

Frisch, M.J., Trucks, G.W., Schlegel, H.B., Scuseria, G.E., Robb, M.A., Cheeseman, J.R., Scalmani, G., Barone, V., Petersson, G.A., Nakatsuji, H., Li, X., Caricato, M., Marenich, A.V., Bloino, J., Janesko, B.G., Gomperts, R., Mennucci, B., Hratchian, H.P., Ortiz, J.V., Izmaylov, A.F., Sonnenberg, J.L., Williams, Ding, F., Lipparini, F., Egidi, F., Goings, J., Peng, B., Petrone, A., Henderson, T., Ranasinghe, D., Zakrzewski, V.G., Gao, J., Rega, N., Zheng, G., Liang, W., Hada, M., Ehara, M., Toyota, K., Fukuda, R., Hasegawa, J., Ishida, M., Nakajima, T., Honda, Y., Kitao, O., Nakai, H., Vreven, T., Throssell, K., Montgomery Jr., J.A., Peralta, J.E., Ogliaro, F., Bearpark, M.J., Heyd, J.J., Brothers, E.N., Kudin, K.N., Staroverov, V.N., Keith, T.A., Kobayashi, R., Normand, J., Raghavachari, K., Rendell, A.P., Burant, J.C., Iyengar, S.S., Tomasi, J., Cossi, M., Millam, J.M., Klene, M., Adamo, C., Cammi, R., Ochterski, J.W., Martin, R.L., Morokuma, K., Farkas, O., Foresman, J.B., and Fox, D.J. (2016). "Gaussian 16 Rev. C.01". (Wallingford, CT).

Hehre, W.J., Radom, L., Schleyer, P.V.R., and Pople, J.A. (1986). *Ab initio molecular orbital theory.* Wiley New York et al.

Kamel, E.M., and Lamsabhi, A.M. (2020). The quasi-irreversible inactivation of cytochrome P450 enzymes by paroxetine: A computational approach. *Organic & Biomolecular Chemistry* 18**,** 3334-3345.

Kamel, E.M., and Lamsabhi, A.M. (2021). Water biocatalytic effect attenuates cytochrome P450-mediated carcinogenicity of diethylnitrosamine: A computational insight. *Organic & Biomolecular Chemistry* 19**,** 9031-9042.

Lee, C., Yang, W., and Parr, R.G. (1988). Development of the Colle-Salvetti correlation-energy formula into a functional of the electron density. *Physical review B* 37**,** 785.

Pettersen, E.F., Goddard, T.D., Huang, C.C., Couch, G.S., Greenblatt, D.M., Meng, E.C., and Ferrin, T.E. (2004). UCSF Chimera—a visualization system for exploratory research and analysis. *Journal of computational chemistry* 25**,** 1605-1612.

Trott, O., and Olson, A.J. (2010). AutoDock Vina: improving the speed and accuracy of docking with a new scoring function, efficient optimization, and multithreading. *Journal of computational chemistry* 31**,** 455-461.
